# Integrative pan-cancer analysis of dipeptidyl peptidase 4 with clinical and *in vitro* validation in prostate cancer

**DOI:** 10.3389/fimmu.2026.1616889

**Published:** 2026-03-12

**Authors:** Yuanan Li, Bingnan Lu, Zihui Zhao, Ming Gong, Donghao Lyu, Haoyu Zhang, Runzhi Huang, Yuntao Yao, Yifan Liu, Ping Huang, Xiuwu Pan

**Affiliations:** 1 Department of Urology, Xinhua Hospital Affiliated to Shanghai Jiao Tong University School of Medicine, Shanghai, China; 2 Department of Urology, Seventh People’s Hospital of Shanghai University of Traditional Chinese Medicine, Shanghai, China; 3 Department of Burn Surgery, The First Affiliated Hospital of Naval Medical University, Shanghai, China

**Keywords:** biomarker, DPP4, immunotherapy, pan-cancer, prostate cancer

## Abstract

**Background:**

Dipeptidyl peptidase 4 (DPP4) plays diverse physiological roles, but its pan-cancer significance and immunomodulatory functions remain poorly characterized.

**Methods:**

We performed an integrative pan-cancer analysis of DPP4, incorporating transcriptomic, genomic, and immunogenomic approaches. Differential expression, ceRNA networks, protein interactions, immune infiltration, drug sensitivity, molecular docking, and molecular dynamics were systematically evaluated. Single-cell sequencing analysis, virtual knockout analysis and TIDE analysis were conducted to validate the role of DPP4 in prostate cancer. Further clinical validation was conducted in a prostate cancer cohort (n=97) using immunohistochemistry, Kaplan-Meier survival analysis, and clinicopathological correlation studies were also conducted. DPP4 expression was assessed by qPCR in 22Rv1 and C4–2 cells treated with dasatinib or midostaurin at IC_50_ concentrations.

**Results:**

DPP4 exhibited tumor-specific dysregulation across multiple cancer types. Its expression correlated significantly with patient prognosis, tumor stage, genomic alterations, immune cell composition, and therapeutic response. In prostate cancer, DPP4 was markedly downregulated (*p* < 0.001) and higher expression predicted better overall survival (*p* < 0.001) and progression-free survival (*p* < 0.001). Significant associations were observed with Gleason score (*p* = 0.03) and WHO/ISUP grade (*p* = 0.03). After dasatinib treatment, DPP4 expression in C4–2 was significantly elevated (*p* < 0.001). On the contrary, DPP4 expression in both 22Rv1 and C4–2 was reduced after treatment with midostarin (*p* < 0.05).

**Conclusion:**

Our study establishes DPP4 as a multifaceted pan-cancer biomarker with prognostic significance and immunotherapeutic implications, particularly in prostate cancer.

## 1 Introduction

Dipeptidyl peptidase 4 (DPP4)/CD26, a 110-kDa membrane-binding protein expressed in various cell types throughout the body, has the function of cleaving N-terminal dipeptides from peptides with a proline or alanine in the penultimate position (1, 2). Previous studies have found that DPP4 plays a significant role in the progression and development of cancer, such as migration, invasion, metastasis, apoptosis, and sensitivity to chemotherapy (3). It has been shown to promote tumor development *in vitro* and in animal models of chronic myeloid leukemia (CML) (4), melanoma (5), hepatocellular carcinoma (HCC) (6, 7), breast cancer (6), and multiple myeloma (8). Conversely, DPP4 enhances tumor suppression *in vitro* and in animal models of colorectal cancer (CRC) (9) and lung cancer (10). While DPP4 has been extensively studied in various malignancies, its functional significance in prostate cancer (PCa) remains particularly compelling yet incompletely understood. Wesley et al. reported that DPP4 inhibits the malignant phenotype of PCa cells by blocking the basic fibroblast growth factor signaling pathway (11), and other studies have also confirmed that DPP4 suppresses tumor growth in PCa (12). However, most existing research lacks a comprehensive comparative perspective across different cancer types, which is essential for defining the specific diagnostic and prognostic value of DPP4.

In our research, we performed a pan-cancer analysis to assess the differential expression, competing endogenous RNA (ceRNA) network, and mutational patterns of DPP4. What’s more, our study highlights its impact on prognosis, immune infiltration patterns, drug sensitivity, molecular docking, and the enrichment of molecular pathways. At last, we conducted a retrospective analysis on 97 patients with prostate cancer at Xinhua Hospital, which included immunohistochemical (IHC) staining of DPP4 in patient samples to evaluate its prognostic relevance.

## 2 Materials and methods

### 2.1 Data acquisition and preprocessing

The retrospective cohort comprised 97 patients diagnosed with prostate cancer at Xinhua Hospital, affiliated with Shanghai Jiao Tong University School of Medicine. The study protocol received formal approval from the institutional Ethics Committee (XHEC-D-2024-145) and adhered to the ethical principles of the Declaration of Helsinki. Written informed consent was obtained from all participants prior to their inclusion. Eligible patients included those who underwent surgery between January 2018 and December 2022 and were diagnosed with histopathological examination. Clinical follow-up was conducted until January 2024. The exclusion criteria included: (1) chronic diseases such as diabetes; (2) severe mental illness; and (3) the presence of other primary tumors.

Tumor and normal tissue samples from these patients were analyzed for DPP4 expression through immunohistochemical (IHC) staining and scoring. Expression data for DPP4 and clinical data across 33 tumor types were gained from the cancer genome atlas (TCGA) database (https://tcga-data.nci.nih.gov/tcga/) (13). **Supplementary Table 1** lists all 33 tumor types and their abbreviations. The data on microRNA (miRNA) were obtained from several sources: miRDB (https://mirdb.org/) (14), miRcode (15), miRWalk (mirwalk.umm.uni-heidelberg.de) (16), DIANA-microT (http://diana.imis.athena-innovation.gr/DianaTools/index.php?r=microtv4/index/) (17), and the Encyclopedia of RNA Interactomes (ENCORI) databases (https://rnasysu.com/encori/) (18). Additionally, miRNA-lncRNA interaction data were extracted from the ENCORI database as well. Protein-protein interaction (PPI) data were sourced from the STRING database (https://cn.string-db.org/) (19), which calculated the combined confidence scores between proteins from multiple sources including experimental data, curated databases, text mining, gene co-expression, gene neighborhood and so on. Microsatellite instability (MSI) scores were derived from the study by Sameek Roychowdhury et al. (20). Data on immune cell infiltration were obtained from TIMER 2.0 (https://timer.cistrome.org/) for estimating immune cell infiltration (21). Cytotoxic T lymphocyte (CTL) infiltration and T cell dysfunction data were retrieved from the Tumor Immune Dysfunction and Exclusion (TIDE) database (http://tide.dfci.harvard.edu/) (22). Drug sensitivity data were sourced from The Cancer Therapeutics Response Portal (CTRP) (https://portals.broadinstitute.org/ctrp/) (23), the Genomics of Drug Sensitivity in Cancer (GDSC) (https://www.cancerrxgene.org/) (24), and the CellMiner databases (https://discover.nci.nih.gov/cellminer/) (25). Signaling pathway data were accessed from the Kyoto Encyclopedia of Genes and Genomes (KEGG) (https://www.genome.jp/kegg/) (26). Single-cell sequencing data was obtained from The Tumor Immune Single Cell Hub 2 (TISCH2) database (https://tisch.compbio.cn) (27), IMMUcan SingleCell RNAseq Database (https://immucanscdb.vital-it.ch/) and Gene Expression Omnibus (GEO) (GSE141445 (28), GSE185344 (29), GSE137829 (30), GSE150692 (31), GSE172301 (32), GSE172316 (32), GSE176031 (33), GSE181294 (34)).

### 2.2 Pan-cancer differential expression analysis

Transcriptome data of DPP4 from The Cancer Genome Atlas (TCGA) underwent analysis utilizing the R “*ggpubr*” package, contrasting expression levels between tumor and normal tissues via the Wilcoxon test. The outcomes were visualized through boxplots.

### 2.3 Pan-cancer ceRNA and protein-protein interaction network construction

Potential miRNAs that target DPP4 were identified through the miRDB, miRcode, miRWalk, and DIANA-microT databases. Key miRNAs were determined by finding the intersection of all potential candidates. Using these key miRNAs, we investigated miRNA-lncRNA interactions within the ENCORI database. The relationship between these key miRNAs and DPP4 across various cancers was also validated using the ENCORI database. The ceRNA network was subsequently visualized with the help of Cytoscape and Sankey diagrams.

On the proteomic level, potential protein interactions involving DPP4 were identified using the STRING database. A PPI network was then constructed to illustrate these interactions.

### 2.4 Pan-cancer clinical relevance analysis

Clinical data were sourced from TCGA database. All raw data were normalized and analyzed in R. The cohort was categorized into high and low DPP4 expression groups based on the median expression level. The correlation between DPP4 expression and survival outcomes, including overall survival (OS), disease-free survival (DFS), disease-specific survival (DSS), and progression-free survival (PFS), were analyzed using the R packages “*survminer*” and “*survival*.” Kaplan-Meier (K-M) survival analysis was conducted to assess the association between DPP4 expression and patients’ survival. Additionally, univariate Cox regression analysis was performed to determine the hazard ratios (HR) for DPP4 expression across 33 different tumor types for OS, DFS, PFS, and DSS, with results displayed in forest plots (35). The relationship between clinical stages of 33 tumor types and DPP4 expression was examined using the Wilcoxon test. Furthermore, we investigated the correlation between bone metastasis and DPP4 expression in four tumor types (BLCA, BRCA, MESO, and PRAD), and visualized the differences in expression values between primary tumors and bone metastases with boxplots. For BRCA, the subtypes (Basal-like, Her-2 enriched, Normal-like, Luminal A, and Luminal B) were categorized according to PAM50 (36). The association between these subtypes and DPP4 expression was analyzed and visualized with boxplots.

### 2.5 Pan-cancer genetic alteration and mutation analysis

The “*maftools*” R package was employed to compute tumor mutational burden (TMB) across 33 cancer types using TCGA whole-exome sequencing data (37). Microsatellite instability (MSI) scores were sourced from the study by Sameek Roychowdhury et al. (20). The relationship between DPP4 expression and both TMB and MSI for each cancer type was assessed through Spearman correlation analysis, and the results were displayed using radar charts.

### 2.6 Pan-cancer ESTIMATE algorithm analysis

The ESTIMATE algorithm was employed to compute microenvironment scores, encompassing immune scores and stromal scores, across 33 tumor types (38). A higher stromal score indicated a greater presence of stromal components, while higher immune scores signified increased infiltration of immune cells. The correlation between DPP4 expression and these two scores was then evaluated using Spearman correlation analysis. Notably, as the abundance of immune cells and stromal cells increased, tumor purity decreased.

Subsequently, the CIBERSORT algorithm, known for its ability to characterize the cell composition of complex tissues based on gene expression profiles, was utilized to estimate the percentage of immune cells in tumor tissues across the same 33 tumor types (39). The Spearman correlation test was applied to examine the relationship between DPP4 expression and the infiltration of specific types of immune cells.

### 2.7 Pan-cancer immune analysis

A total of 47 immune-related genes were compiled for analysis (40). Spearman correlation analysis was employed to construct a co-expression heatmap, illustrating the relationship between the expression levels of these immune genes and DPP4.

For immune infiltration estimation, TIMER 2.0 (https://timer.cistrome.org/) was utilized (21). Spearman correlation analysis was then conducted to explore the association between DPP4 expression and immune cell infiltration, with the findings visualized using heatmaps.

### 2.8 Pan-cancer gene set enrichment analysis of DPP4

To investigate the DPP4-related signaling pathways, gene set enrichment analysis (GSEA) was conducted across 33 tumor types using the “*clusterProfiler*” R package (41). Patient samples from TCGA were categorized into high and low DPP4 expression groups based on the median DPP4 expression. Subsequently, we compared the gene expression profiles of each patient’s sample with the gene expression profiles of specific KEGG pathways. The top five KEGG pathways with the highest scores and *p* < 0.05 in each tumor type were visualized.

### 2.9 Pan-cancer drug sensitivity prediction

Data on the half maximal inhibitory concentration (IC_50_) values of small molecules and the corresponding mRNA expression levels of DPP4 in specific cell lines from the GDSC, CTRP, and CellMiner databases were collected. Pearson correlation analysis was performed to examine the correlation between DPP4 expression and the IC_50_ values of small molecule drugs in the CTPR and GDSC databases. Similarly, in the CellMiner database, Pearson correlation analysis was conducted to investigate the relationship between DPP4 expression and the Z scores of specific drugs. Higher Z scores indicated increased drug sensitivity.

Molecular docking analysis, a computational technique that predicts the binding affinity of ligands to receptor proteins, has been widely applied in studying protein-ligand interactions and predicting binding affinities, as demonstrated in previous research (42, 43). The 3D structure of DPP4 was obtained from the PDB database with the PDB ID 1r9m, and processed using PyMOL (Version 1.3, Schrödinger, LLC) to eliminate solvent molecules, non-target proteins, and ligands. A total of four drugs were selected as ligands for molecular docking with DPP4, the 3D structures of which were downloaded from PubChem (dasatinib, PubChem CID 3062316; midostaurin, PubChem CID 9829523; saracatinib, PubChem CID 10302451; and selumetinib, PubChem CID 10127622). AutoDock Tools was then used to add hydrogen atoms and generate PDBQT files for both the receptors and ligands (44, 45), which also facilitated the setup of a 3D grid box around the receptor’s binding site. Subsequently, AutoDock Vina 1.1.2 was utilized to predict the optimal binding conformations between the ligands and receptors, and the docking results were analyzed in PyMOL (46). During this process, it employed an efficient algorithm that combined a global optimization strategy, specifically the Iterated Local Search (ILS) global optimizer, with gradient-based local optimization using the Broyden-Fletcher-Goldfarb-Shanno (BFGS) method to predict the binding modes of small molecules to their protein targets (46). The number of independent runs was set as 40 in our docking experiment. DoGSiteScorer tool was used to analyze and identify potential binding pockets, providing the coordinates and radius of each pocket (47, 48). The unit of binding energy was kcal/mol in our study.

Subsequently, to validate the stability of the docking results, all-atom molecular dynamics (MD) simulations were conducted for each of the four drug-DPP4 complexes exhibiting the lowest binding energies using GROMACS 2022. Prior to simulation setup, the optimal docked complex structures were preprocessed by removing redundant water molecules and non-specifically bound small-molecule impurities from the receptor protein DPP4. Topology files for the receptor protein were generated using the pdb2gmx tool with the CHARMM36 force field (49), while the ligands were parameterized using the CGenFF force field through the AutoFF web server; all generated parameters were rigorously validated to ensure reasonable bond lengths, angles, and dihedral angles.

Each complex system was centered in the simulation box using the editconf tool and solvated in a cubic TIP3P water box extending 1.0 nm from the solute (50) to fully enclose the complex and prevent unphysical solute–boundary interactions. The systems were then neutralized by replacing selected water molecules with Na^+^/Cl^-^ ions using the gmx genion tool (ion types and numbers determined by net charge), with the ionic strength adjusted to 0.15 mol/L to mimic physiological conditions and enhance the physiological relevance of the simulations. Short-range van der Waals and electrostatic interactions were truncated at 1.0 nm, while long-range electrostatics were treated using the Particle Mesh Ewald (PME) method. Bonds involving hydrogen atoms were constrained using the SHAKE algorithm, and the leap-frog integrator was employed with a 1 fs time step.

Prior to the production run, multistage energy minimization was conducted to eliminate local steric clashes: (1) the solute was restrained while the solvent was minimized; (2) counterions were restrained for a second minimization; and (3) the entire unrestrained system was globally minimized. Each stage consisted of 3000 steps of steepest descent followed by 2000 steps of conjugate gradient optimization.

The production simulation was carried out for 100 ns in the NPT ensemble at a physiological temperature of 310 K. Trajectory frames were extracted at regular intervals, and post-simulation analyses of RMSD, root-mean-square fluctuation (RMSF), hydrogen bond numbers (H-bonds), radius of gyration (Rg), and solvent-accessible surface area (SASA) were performed using the gmx rms, gmx rmsf, gmx hbond, gmx gyrate, and gmx sasa tools, respectively. All data were subsequently processed and visualized using GraphPad Prism and PyMOL 2.5 to illustrate the conformational stability, residue flexibility, and non-covalent interaction features of the drug-DPP4 complexes.

### 2.10 Single-cell sequencing analysis in prostate cancer

To investigate DPP4 expression at the single-cell level, we first explored the TISCH database for DPP4 expression in prostate cancer. Then, we queried the IMMUcan SingleCell RNAseq Database, which profiles gene expression across diverse cell types in pan-cancer single-cell datasets. Subsequently, scRNA-seq datasets were acquired from the Gene Expression Omnibus (GEO) (accession number: GSE141445 (28), GSE185344 (29), GSE137829 (30), GSE150692 (31), GSE172301 (32), GSE172316 (32), GSE176031 (33), GSE181294 (34)). Subsequent to quality control, the data were processed using the Seurat R package, involving multi-sample integration, log-normalization, feature scaling, and the identification of highly variable genes (51). To mitigate batch-effects across the diverse datasets, the *Harmony* algorithm was implemented to generate a batch-corrected expression matrix (52). Cellular clustering was achieved by constructing a shared nearest-neighbor graph (using the first 15 Harmony-corrected dimensions). High-dimensional data were then projected into two-dimensional space using Uniform Manifold Approximation and Projection (UMAP) for visualization. Cell lineages were manually annotated based on cluster-specific markers identified through the *FindMarkers* function. Furthermore, intercellular signaling patterns were reconstructed using the *CellChat* R package (53), utilizing the curated *CellChatDB* to quantify and visualize ligand-receptor interaction strengths across cell types.

### 2.11 Virtual knockout analysis of DPP4

To evaluate the transcriptomic consequences of DPP4 perturbation, we conducted virtual knockout (KO) experiments using *scTenifoldKnk*, a machine learning-based framework designed for scRNA-seq data. Genes exhibiting significant perturbation (adjusted *p* < 0.05) underwent functional enrichment analysis, while the entire gene list was subjected to Gene Oncology (GO) to elucidate the biological pathways modulated by DPP4 loss. To further explore the functional architecture of these perturbed genes, a protein-protein interaction (PPI) network was constructed and visualized using the STRING database (https://cn.string-db.org/) (19).

### 2.12 TIDE analysis in prostate cancer

The TIDE database was utilized to investigate the correlation between DPP4 expression and CTL (22), so as to predict the response to immunotherapy. Expression profile was retrieved from TCGA-PRAD database, and after normalization, it was uploaded to TIDE database for response prediction. All patients were divided into high- and low-DPP4 expression groups depending on the median of DPP4 expression, and its correlation between response rate for immune checkpoint blockade therapy, CTL infiltration, TIDE score, rate of tumor T cell dysfunction potential, and rate of tumor T cell exclusion potential were explored and visualized.

### 2.13 Immunohistochemical analysis and grading

Our retrospective cohort consists of all prostate cancer patients who underwent surgical treatment at our institution between August 2017 and January 2022 and provided informed consent for inclusion. Clinical and pathological data were extracted from patient medical records and pathology reports, including Gleason score, TNM stage, and treatment history (radiotherapy, chemotherapy, endocrine therapy, and immunotherapy). Structured telephone follow-ups were conducted to collect additional information regarding pre- and post-operative medication use, longitudinal PSA measurements, biochemical recurrence, clinical recurrence or metastasis, and overall survival status. To minimize potential bias, patients with incomplete or missing key clinical and pathological information were excluded.

Tumor and normal tissue samples from 97 individuals diagnosed with prostate cancer were initially fixed, embedded in paraffin, and sliced. These samples underwent a process of dewaxing and rehydration, followed by antigen retrieval for DPP4 using citrate buffer (pH = 6.0). To block endogenous hydrogen peroxide, the samples were treated with 3% hydrogen peroxide solution and washed with PBS. To prevent non-specific antigen binding, 3% bovine serum albumin (BSA) was applied. Primary antibodies against human DPP4 (1:200; Affinity Biosciences Cat# DF12387, RRID: AB_2845192) were then introduced, followed by secondary antibodies conjugated with horseradish peroxidase (HRP). Color development was facilitated by adding diaminobenzidine (DAB), a specific chromogen for HRP, which produces a visible color change when DPP4 is present. Hematoxylin was also used to enhance contrast and accentuate the nuclei. The samples were then dehydrated using a graded series of alcohol solutions and visualized under a light microscope. Two qualified pathologists in prostate cancer were consulted to evaluate the slides, and conflicted ones were arbitrated by a third pathologist.

The IHC scoring criteria were based on the H-score system: H-score = ∑(pi×i) = (percentage of weak intensity*100) + (percentage of moderate intensity*200) + (percentage of strong intensity*300) (54). The IHC scores ranged from 0 to 300. Paired t-test was conducted to compare the expression of DPP4 between tumor and normal tissues.

### 2.14 Clinical analysis of DPP4 in prostate cancer

Demographic, tumor characteristic data and survival outcomes were collated for the cohort of 97 prostate cancer patients. Utilization of the R software’s “*surv_cutpoint*” function facilitated the determination of an optimal cutoff point for IHC scores. K-M survival curves were constructed to delineate differences in OS and PFS, with statistical significance analyzed via the Log-rank test. Multivariate Cox regression analysis was further conducted, visualized with a forest plot.

### 2.15 *In vitro* validation of DPP4 in prostate cancer

Human prostate cancer cell lines 22Rv1 and C4–2 were obtained from the American Type Culture Collection (ATCC). 22Rv1 cells were cultured in RPMI-1640 medium (Gibco, C11875500BT) supplemented with 20% fetal bovine serum (FBS; Gibco, A5256701) and 1% penicillin–streptomycin (Pen/Strep; Gibco, 15140122). C4–2 cells were cultured in DMEM/F12 medium (Gibco, 21331-020) containing 10% FBS (Gibco, A5256701) and 1% Pen/Strep (Gibco, 15140122).

For half-maximal inhibitory concentration (IC_50_) determination, 1,000 cells per well were seeded into 96-well plates and allowed to adhere for 24 h before drug exposure. Dasatinib was administered to 22Rv1 cells at concentrations of 10, 50, 100, 500, and 1,000 µM, and to C4–2 cells at concentrations of 0.1, 5, 10, 50, and 200 µM. Midostaurin was administered to both 22Rv1 and C4–2 cells at concentrations of 0.1, 1, 5, 50, and 200 µM. Following 24h of treatment, cell viability was assessed using the Cell Counting Kit-8 (CCK-8) assay. Briefly, the culture medium was replaced with 90 µl of fresh medium containing 10 µl of CCK-8 reagent per well and incubated for 2 h. Absorbance was measured at 450 nm, and dose–response curves were generated to calculate IC_50_ values using GraphPad Prism software.

Subsequently, cells were treated with dasatinib or midostaurin at their respective IC_50_ concentrations, with dimethyl sulfoxide (DMSO)-treated cells serving as controls. After 24 h of incubation, total RNA was extracted, and quantitative PCR (qPCR) was performed to evaluate DPP4 mRNA expression. Relative expression levels were calculated using the 2^-ΔΔCt^ method, with GAPDH as the internal reference. Two pairs of primers were used for DPP4 amplification:

The primer of DPP4 was:

F: 5-GGGTCACATGGTCACCAGTG-3, R: 5-TCTGTGTCGTTAAATTGGGCATA-3.

F: 5-AGTGGCACGGCAACACATT-3, R: 5-AGAGCTTCTATCCCGATGACTT-3.

### 2.16 Statistical analysis

Statistical analysis was performed using R version 4.3.2. Categorical data were represented as frequencies and percentages, while continuous data were represented as means with standard deviations or medians with interquartile ranges. A two-tailed p-value of less than 0.05 and a false discovery rate (FDR) of less than 0.05 were considered statistically significant.

## 3 Results

### 3.1 Pan-cancer transcriptome and proteome analysis

**Figure 1:**
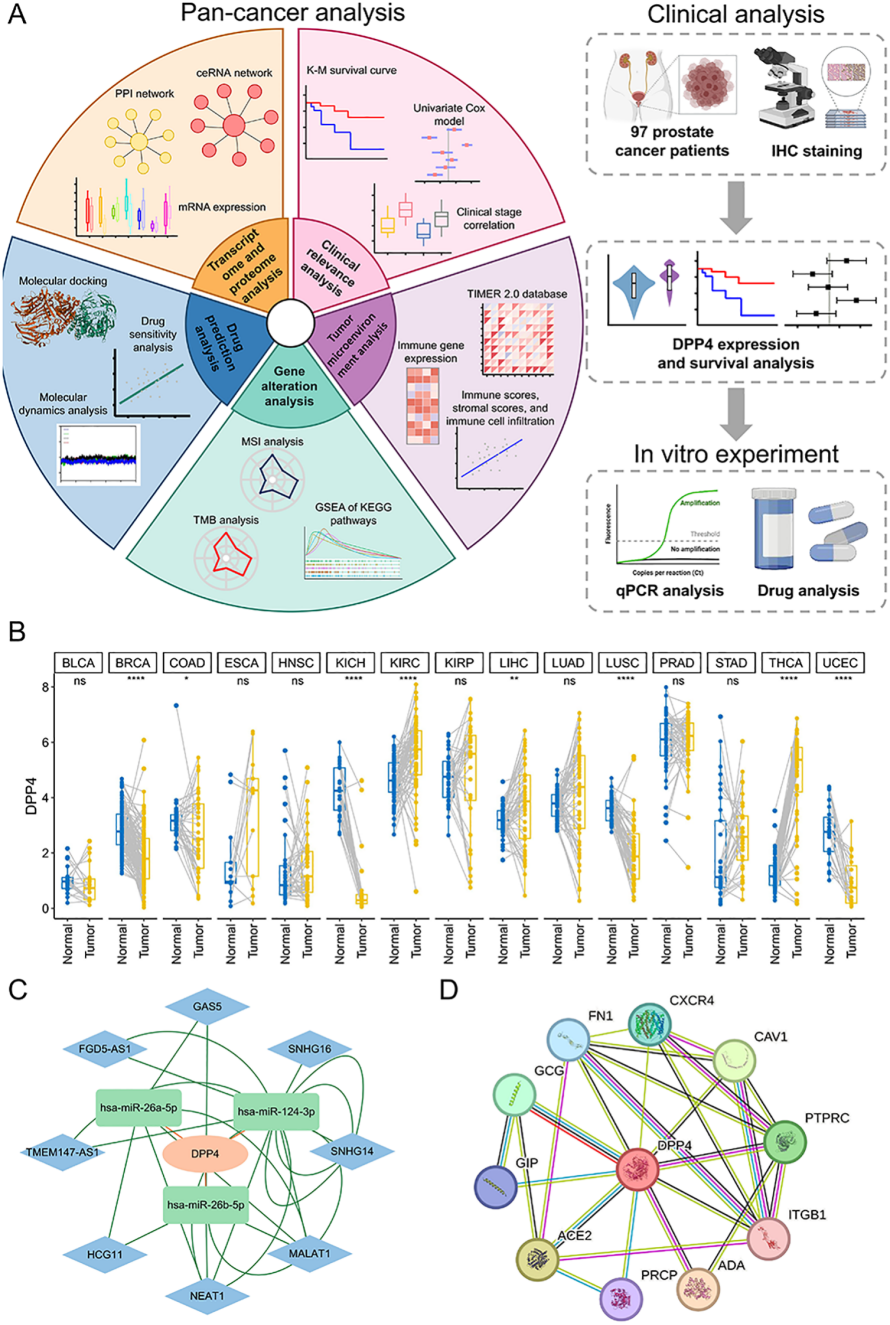
The flow chart, differential expression analysis, ceRNA network construction, and PPI network construction. **(A)** The flow chart of our analysis. **(B)** Expression of DPP4 in 33 tumor types between normal tissues and tumor tissues. DPP4 expression was down-regulated in BRCA (*p* < 0.001), CESC (*p* < 0.05), CHOL (*p* < 0.001), COAD (*p* < 0.05), KICH (*p* < 0.001), LUSC (*p* < 0.001), PCPG (*p* < 0.05), READ (*p* < 0.01) and UCEC (*p* < 0.01), while it was up-regulated in GBM (*p* < 0.01), KIRC (*p* < 0.001), KIRP (*p* < 0.001), LIHC (*p* < 0.001), LUAD (*p* < 0.001), STAD (*p* < 0.05) and THCA (*p* < 0.001). **(C)** The ceRNA network of DPP4. There were three key miRNAs correlated with DPP4 and there were eight lncRNAs correlated with the key miRNAs. **(D)** The PPI network of DPP4. At the proteomic level, DPP4 was closely associated with FN1, CXCR4, CAV1, ITGB1, PTPRC, ADA, GCG, GIP, ACE2, and PRCP. ceRNA, competing endogenous RNA; lncRNA, long non-coding RNA; PPI, Protein-Protein Interaction. *p < 0.05, **p < 0.01, ***p < 0.001.

**Figure 1A** and **Supplementary Figure 1** were the analysis flow charts of our study. **Figure 1B** displays the differential expression analysis of DPP4 expression between tumor and normal tissues. It revealed that DPP4 expression was reduced in BRCA, CESC, CHOL, COAD, KICH, LUSC, PCPG, READ, and UCEC, whereas it was elevated in GBM, KIRC, KIRP, LIHC, LUAD, STAD, and THCA. To further explore the mechanisms regulating DPP4, we examined possible miRNA interactions and constructed a ceRNA network. We cross-referenced target miRNAs for DPP4 from the miRDB, miRcode, miRWalk, and DIANA-microT databases, pinpointing key miRNAs such as hsa-miR-124 and hsa-miR-26 (**Supplementary Figure 2A**). Subsequently, we identified lncRNAs targeted by these key miRNAs (hsa-miR-124-3p, hsa-miR-26a-5p, and hsa-miR-26b-5p) and created a ceRNA network to illustrate the DPP4-miRNA-lncRNA interactions (**Figure 1C**, **Supplementary Figure 2B**). We also confirmed that DPP4 expression in various cancers was significantly correlated with the expression levels of these three key miRNAs (**Supplementary Figure 2C**). Furthermore, an analysis of DPP4’s protein-protein interactions revealed close associations with FN1, CXCR4, CAV1, ITGB1, PTPRC, ADA, GCG, GIP, ACE2, and PRCP, and a PPI network was constructed (**Figure 1D**). These findings suggest potential roles of DPP4 in cancer through its interactions at multiple molecular levels.

### 3.2 Pan-cancer clinical relevance analysis

**Figure 2:**
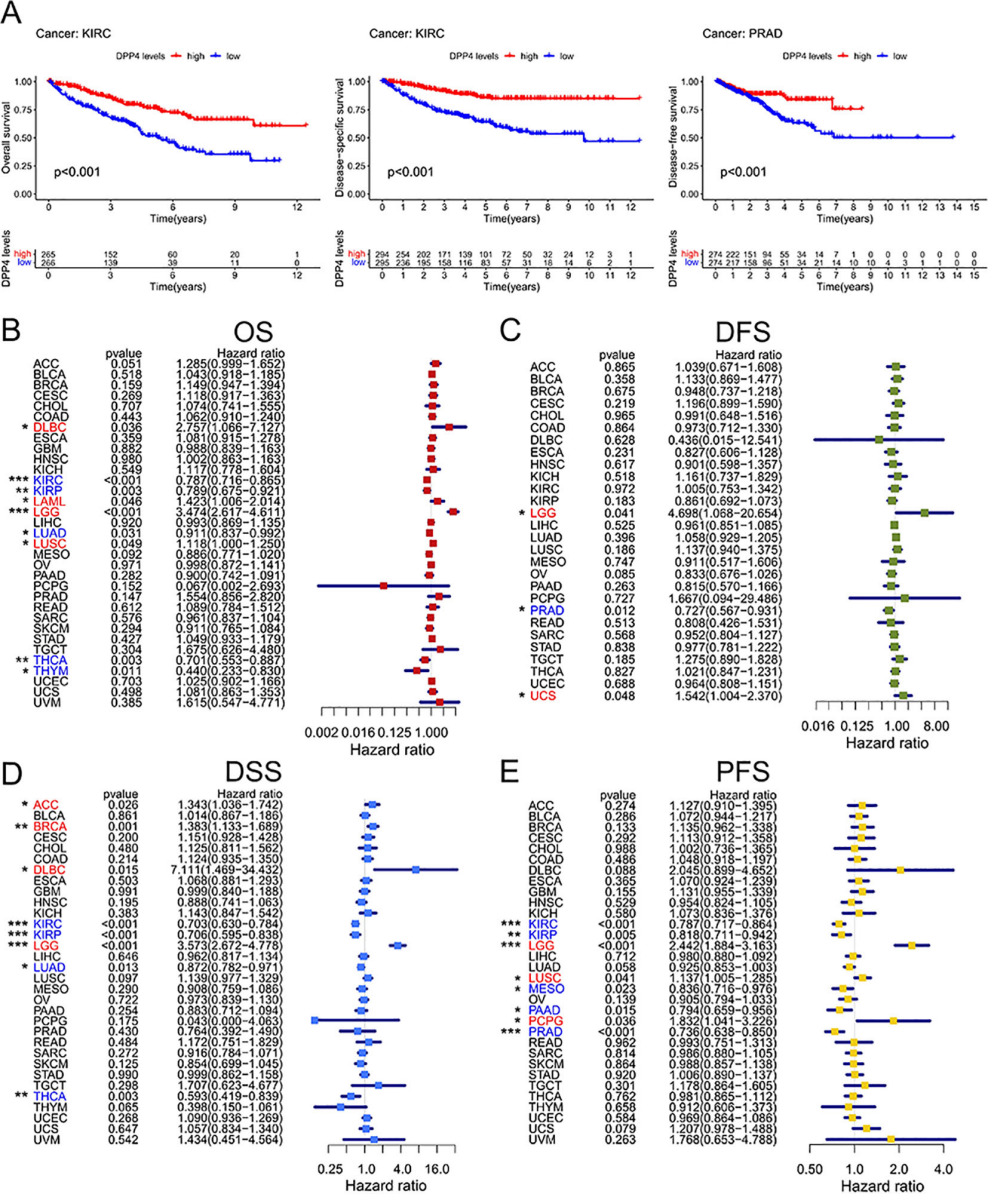
Survival analysis and univariate Cox regression analysis in pan-cancer. **(A)** Survival analysis of DPP4 in pan-cancer. K-M survival curves indicated DPP4 was positively correlated with OS in KIRC (*p* < 0.001), with DSS in KIRC (*p* < 0.001), and with DFS in PRAD (*p* < 0.001). **(B)** Univariate Cox model of OS. DPP4 expression was a risk factor in DLBC (HR = 2.757, 95%CI = 1.066-7.127, *p* = 0.036), LAML (HR = 2.757, 95%CI = 1.066-7.127, *p* = 0.036), LGG (HR = 3.474, 95%CI = 2.617-4.611, *p* < 0.001), and LUSC (HR = 1.118, 95%CI = 1.000-2.014, *p* < 0.049), and it was a protective factor in KIRC (HR = 0.787, 95%CI = 0.716-0.865, *p* < 0.001), KIRP (HR = 0.789, 95%CI = 0.675-0.921, *p* = 0.003), LUAD (HR = 0.911, 95%CI = 0.837-0.992, *p* = 0.031), THCA (HR = 0.701, 95%CI = 0.553-0.887, *p* = 0.003), and THYM (HR = 0.440, 95%CI = 0.233-0.830, *p* = 0.011). **(C)** Univariate Cox model of DFS. DPP4 expression was a risk factor in LGG (HR = 4.698, 95%CI = 1.068-20.654, *p* = 0.041) and UCS (HR = 1.542, 95%CI = 1.004-2.370, *p* = 0.048), while it was a protective factor in PRAD (HR = 0.727, 95%CI = 0.567-0.931, *p* = 0.012). **(D)** Univariate Cox model in DSS. DPP4 expression was a risk factor in LGG (HR = 3.573, 95%CI = 2.672-4.778, *p* < 0.001), BRCA (HR = 1.383, 95%CI = 1.133-1.689, *p* = 0.001), DLBC (HR = 7.111, 95%CI = 1.469-34.432, *p* = 0.015), and ACC (HR = 1.343, 95%CI = 1.036-1.742, *p* = 0.026), while it was a protective factor in KIRC (HR = 0.703, 95%CI = 0.630-0.784, *p* < 0.001), KIRP (HR = 0.706, 95%CI = 0.595-0.838, *p* < 0.001), THCA (HR = 0.593, 95%CI = 0.419-0.839, *p* = 0.003), and LUAD (HR = 0.872, 95%CI = 0.782-0.971, *p* = 0.013). **(E)** Univariate Cox model in PFS. DPP4 expression was a risk factor in LGG (HR = 2.442, 95%CI = 1.884-3.163, *p* < 0.001), PCPG (HR = 1.832, 95%CI = 1.041-3.226, *p* = 0.036), and LUSC (HR = 1.137, 95%CI = 1.005-1.285, *p* = 0.041), while it was a protective factor in KIRC (HR = 0.787, 95%CI = 0.717-0.864, *p* < 0.001), PRAD (HR = 0.736, 95%CI = 0.638-0.850, *p* < 0.001), KIRP (HR = 0.818, 95%CI = 0.711-0.942, *p* = 0.005), PAAD (HR = 0.794, 95%CI = 0.659-0.956, *p* = 0.015), and MESO (HR = 0.836, 95%CI = 0.716-0.976, *p* = 0.023). OS, Overall Survival; DFS, Disease-Free Survival; DSS, Disease-Specific Survival; PFS, Progression-Free Survival. *p < 0.05, **p < 0.01, ***p < 0.001.

To investigate the correlation between DPP4 expression and clinical prognosis, survival analysis was conducted between high and low expression groups. K-M survival curves indicated that DPP4 expression was negatively correlated with OS in BLCA, while positively correlated in KIRC, KIRP, and MESO. In terms of DFS, DPP4 was negatively associated with DFS in LUAD, but positively correlated with DFS in PRAD. For DSS, DPP4 showed a negative correlation with DSS in LUSC, but a positive correlation in KIRC, KIRP, MESO, and SKCM. Regarding PFS, DPP4 was negatively associated with PFS in LGG and LUSC, but positively correlated with PFS in KIRC, MESO, and PRAD (**Figure 2A**, **Supplementary Figure 3**).

Moreover, univariate Cox regression models were developed for OS, DFS, DSS, and PFS. The analysis revealed that DPP4 expression served as a risk factor for OS in DLBC, LAML, LGG, and LUSC, while acting as a protective factor in KIRC, KIRP, LUAD, THCA, and THYM (**Figure 2B**). For DFS, DPP4 expression was identified as a risk factor in LGG and UCS, but a protective factor in PRAD (**Figure 2C**). Regarding DSS, DPP4 expression was a risk factor in LGG, BRCA, DLBC, and ACC, whereas it was a protective factor in KIRC, KIRP, THCA, and LUAD (**Figure 2D**). In terms of PFS, DPP4 expression was a risk factor in LGG, PCPG, and LUSC, but a protective factor in KIRC, PRAD, KIRP, PAAD, and MESO (**Figure 2E**).

What’s more, the correlation between DPP4 expression and clinical stage in pan-cancer was analyzed. DPP4 was positively correlated with the clinical stages of KIRC and TGCT (**Supplementary Figure 4A**). For four tumor types which easily metastasized to bones (BLCA, BRCA, MESO and PRAD), correlation analysis indicated that bone metastasis was not significantly related to DPP4 expression (**Supplementary Figure 4B**). However, the expression of DPP4 was significantly associated with the molecular subtypes of BRCA, and the normal Luminal B type showed the lowest DPP4 expression (**Supplementary Figure 4C**).

### 3.3 Pan-cancer gene alteration analysis

**Figure 3:**
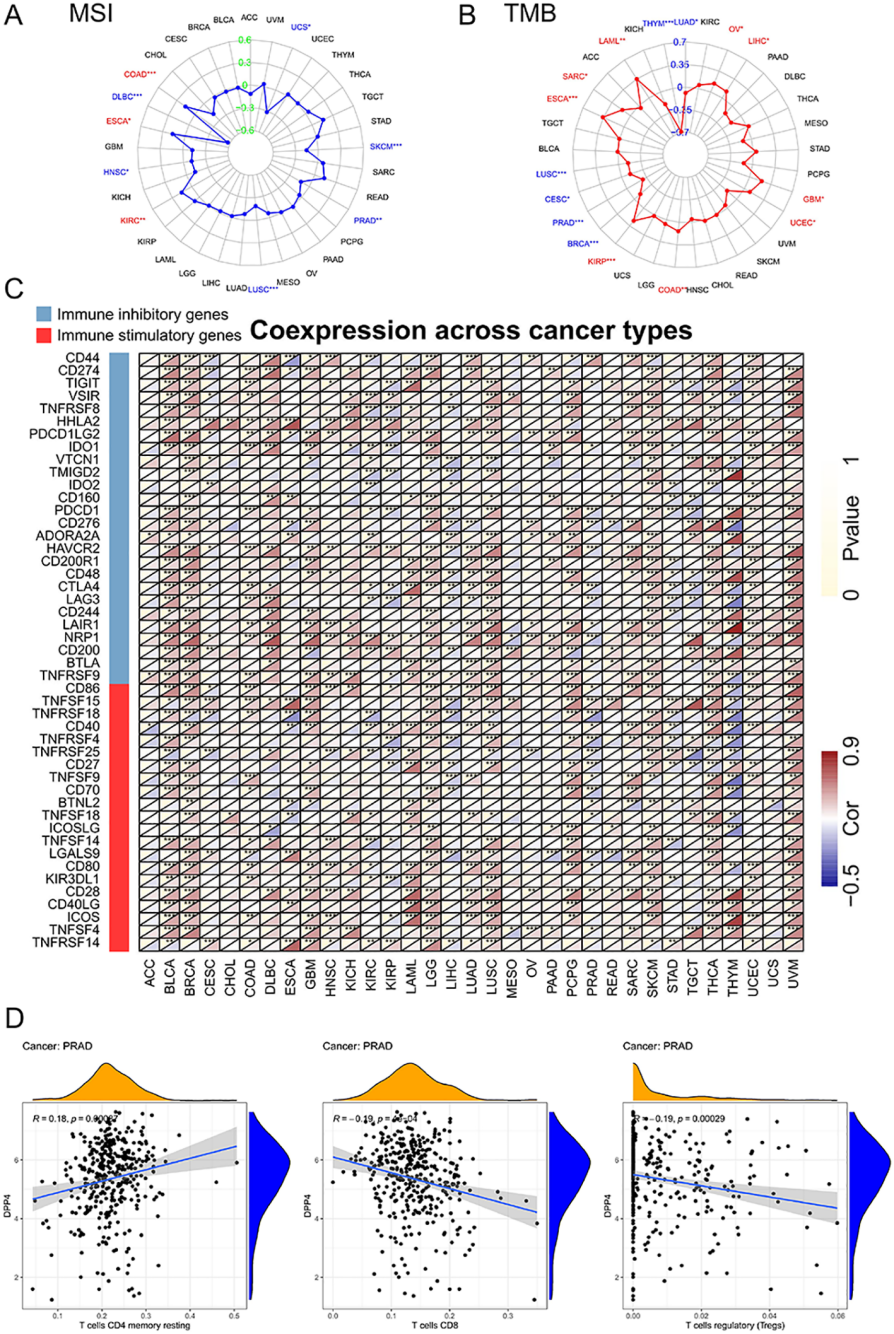
Relationship of DPP4 expression with TME in pan-cancer. **(A)** Correlation of DPP4 expression with MSI. It was positively correlated with MSI in COAD (*p* < 0.001), ESCA (*p* < 0.05), and KIRC (*p* < 0.01), while it was negatively related in DLBC (*p* < 0.001), HNSC (*p* < 0.05), LUSC (*p* < 0.001), PRAD (*p* < 0.01), SKCM (*p* < 0.001), and UCS (*p* < 0.05). **(B)** Correlation of DPP4 expression with TMB. It was positively correlated with TMB in LAML (*p* < 0.01), SARC (*p* < 0.05), ESCA (*p* < 0.001), KIRP (*p* < 0.001), COAD (*p* < 0.01), UCEC (*p* < 0.05), GBM (*p* < 0.05), LIHC (*p* < 0.05), and OV (*p* < 0.05), while it was negatively correlated in THYM (*p* < 0.001), LUSC (*p* < 0.001), CESC (*p* < 0.05), PRAD (*p* < 0.001), BRCA (*p* < 0.001), and LUAD (*p* < 0.05). **(C)** The relationship between DPP4 expression and immune-related genes. There is a significant association between DPP4 expression and immune-related genes across various cancers, particularly with NRP1 and HHLA2. Additionally, the majority of immune genes showed a positive correlation with DPP4 expression in BLCA, BRCA, LGG, SKCM, and THCA. **(D)** In PRAD, DPP4 expression was positively correlated with T cells CD4 memory resting (R = 0.18, *p* < 0.001), while it was negatively correlated with T cells CD8 (R = -0.19, *p* < 0.001), and T cells regulatory (R = -0.19, *p* < 0.001). MSI, Microsatellite Instability; TMB, Tumor Mutation Burden; TME, Tumor Microenvironment. *p < 0.05, **p < 0.01, ***p < 0.001.

Given the importance of TMB and MSI as predictors of immunotherapy response, we investigated the correlation between DPP4 expression and these factors. DPP4 expression showed a negative association with MSI in DLBC, HNSC, LUSC, PRAD, SKCM, and UCS, while a positive correlation was found in COAD, ESCA, and KIRC (**Figure 3A**). Spearman correlation analysis indicated that DPP4 expression was negatively correlated with TMB in several cancer types, including THYM, LUSC, CESC, PRAD, BRCA, and LUAD. Conversely, a positive correlation was observed in LAML, SARC, ESCA, KIRP, COAD, UCEC, GBM, LIHC, and OV (**Figure 3B**).

### 3.4 Pan-cancer tumor microenvironment analysis

To evaluate the relationship between DPP4 expression and TME, the stromal scores and immune scores were calculated with the help of the ESTIMATE algorithm, and their correlation with DPP4 expression was shown. In the aspect of immune score, the expression of DPP4 was positively correlated with immune scores in BLCA, BRCA, COAD, DLBC, GBM, HNSC, LAML, LGG, LUAD, LUSC, OV, PCPG, SARC, SKCM, THCA, THYM, UCEC, and UVM, while it was negatively correlated in KIRC and LIHC. For stromal scores, the expression of DPP4 was positively correlated in BLCA, BRCA, CESC, GBM, HNSC, LGG, LUAD, LUSC, OV, PAAD, PCPG, SARC, SKCM, TGCT, UCEC, and UVM (**Supplementary Figure 5**).

Spearman analysis revealed a significant association between DPP4 expression and immune-related genes across various cancers, particularly with NRP1 and HHLA2. Additionally, the majority of immune genes showed a positive correlation with DPP4 expression in BLCA, BRCA, LGG, SKCM, and THCA (**Supplementary Figure 5**). To further investigate immune infiltration, we employed the CIBERSORT algorithm. In PRAD, DPP4 expression was positively correlated with T cells CD4 memory resting, while it was negatively correlated with T cells CD8, and T cells regulatory. In BLCA, DPP4 expression was negatively correlated with B cells memory, T cells follicular helper, Tregs, while it was positively correlated with macrophages Mo0, macrophages M2, and neutrophils. In BRCA, DPP4 expression was negatively correlated with macrophages M2 and T cells follicular helper, while it was positively correlated with B cells naïve, dendritic cells activated, dendritic cells resting, T cells CD4 memory activated, and T cells CD4 memory resting. In LGG, DPP4 expression was negatively correlated with macrophages M2, mast cells activated, monocytes, and NK cells resting, while it was positively correlated with macrophages M0, macrophages M1, neutrophils, T cells CD4 memory resting, and T cells CD8. In SKCM, DPP4 expression was negatively correlated with macrophages M2, it was positively correlated with dendritic cells resting. In THCA, DPP4 expression was negatively correlated with B cells memory, macrophages M1, and T cells CD, while it was positively correlated with dendritic cells activated, dendritic cells resting, and T cells CD4 memory resting. (**Figure 3C**, **Supplementary Figure 6**). To further elucidate the relationship between DPP4 expression and immune cell infiltration in pan-cancer, we used the TIMER 2.0 database. The results demonstrated that DPP4 expression was positively correlated with cancer-associated fibroblasts (CAF), macrophages, endothelial cells, and neutrophils in most cancer types (**Supplementary Figure 7**).

### 3.5 Pan-cancer functional enrichment analysis

Using GSEA, we investigated the potential signaling pathways associated with DPP4 expression. The KEGG pathways most frequently enriched in high-DPP4 groups included “ascorbate and aldarate metabolism”, “olfactory transduction”, “pentose and glucuronate interconversions”, “porphyrin and chlorophyll metabolism”, and “starch and sucrose metabolism”. To be specific, “Ascorbate and aldarate metabolism” was up-regulated in BRCA, PCPG, and UCEC, while it was down-regulated in LUAD and MESO. “Olfactory transduction” was up-regulated in BRCA and ESCA, while it was down-regulated in OV and STAD. “Pentose and glucuronate interconversions” was up-regulated in BRCA, PCPG, and UCEC, while it was down-regulated in LUAD and MESO. “Porphyrin and chlorophyll metabolism” was up-regulated in BRCA, PCPG, UCEC, and LGG, while it was down-regulated in THYM. “Starch and sucrose metabolism” was up-regulated in UCEC, CESC, and TGCT, while it was down-regulated in THYM (**Supplementary Figure 8**, **Supplementary Figure 9**).

### 3.6 Pan-cancer drug sensitivity prediction analysis

**Figure 4:**
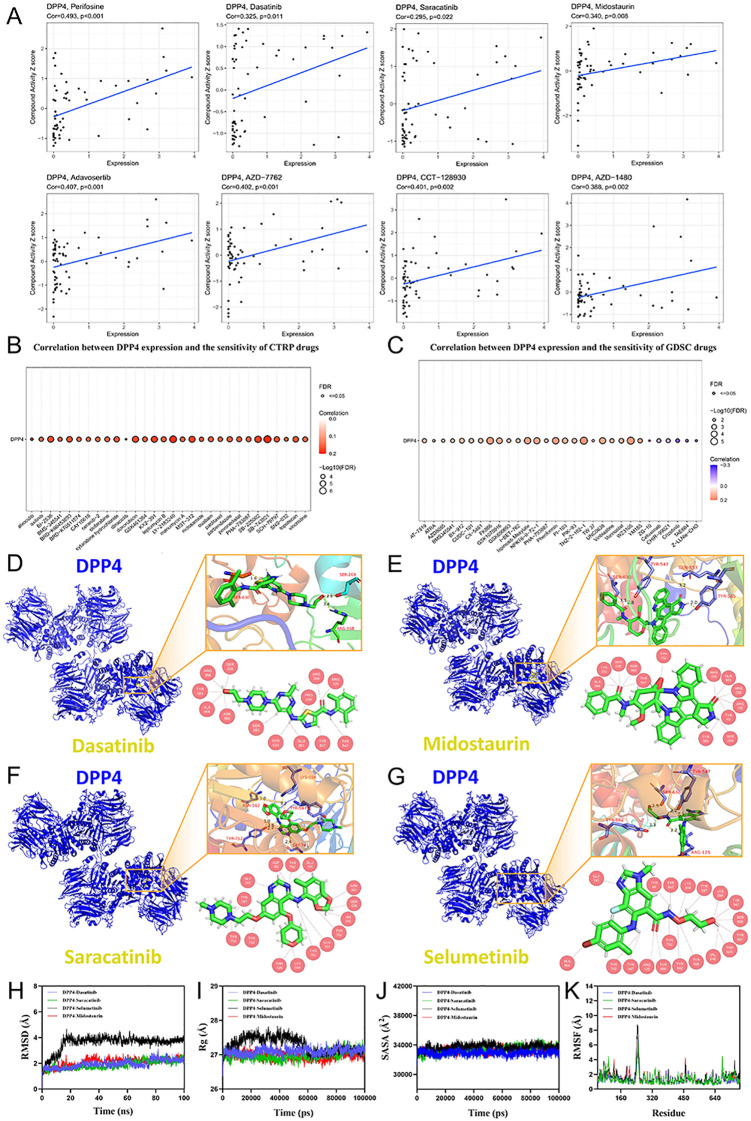
Drug sensitivity prediction for DPP4 in **(A)** CellMiner, **(B)** CTRP and **(C)** GDSC databases. The expression of DPP4 was negatively related with drug sensitivity of most drugs. However, several drugs were positively related with DPP4 expression, including perifosine and adavosertib from CellMiner, dasatinib and saracatinib from CTRP, and cetuximab and crizotinib from GDSC. **(D)** The molecular docking analysis of DPP4 and dasatinib. **(E)** The molecular docking analysis of DPP4 and midostaurin. **(F)** The molecular docking analysis of DPP4 and saracatinib. **(G)** The molecular docking analysis of DPP4 and selumetinib. The possible binding sites were illustrated. **(H)** RMSD values of the protein-ligand complexes over time. The DPP4-Saracatinib complex reached equilibrium after 20 ns, with its RMSD fluctuating around 2.2 Å. The DPP4-Selumetinib complex reached equilibrium after 20 ns, fluctuating around 4.1 Å. The DPP4-Dasatinib complex reached equilibrium after 20 ns, fluctuating around 2.0 Å. The DPP4-Midostaurin complex reached equilibrium after 30 ns, fluctuating around 2.2 Å. **(I)** Rg of the protein-ligand complexes over time. All complex systems exhibited only minor fluctuations throughout the simulation. **(J)** SASA of the protein-ligand complexes over time. The results indicate that the SASA of the complexes did not change significantly after ligand binding to DPP4. **(K)** RMSF of the protein-ligand complexes. The RMSF values for all complexes were relatively low, with most residues fluctuating below 3 Å. RMSD, Root Mean Square Deviation; RMSF, Root-Mean-Square Fluctuation; Rg, Radius of gyration; SASA, Solvent-Accessible Surface Area.

In order to unveil the potential therapeutic value of DPP4 in pan-cancer, we conducted drug sensitivity prediction for DPP4 depending on CellMiner, CTRP, and GDSC databases (**Figures 4A–C**). Higher DPP4 expression was generally linked to reduced drug sensitivity for most drugs. However, certain drugs’ sensitivity showed a positive correlation with DPP4 expression, including perifosine and adavosertib from CellMiner, dasatinib and saracatinib from CTRP, and cetuximab and crizotinib from GDSC. This indicates that DPP4 might influence specific cancer-drug interactions, suggesting that targeting DPP4 could be beneficial in future anti-cancer treatments.

Potential binding pattern between DPP4 and four drugs (dasatinib, midostaurin, saracatinib, and selumetinib) was identified through intersection for further research (**Supplementary Figure 10A**). When dasatinib bond with SER-630, SER-209, and ARG-358, it showed the lowest affinity of -8.8 kcal/mol (**Figure 4D**, **Supplementary Figure 10B**). When midostaurin bond with SER-630, TYR-547, GLN-553 and TYR-585, the lowest affinity of -10.2 kcal/mol was obtained (**Figure 4E**). When saracatinib bond with LYS-554, TYR-547, ASN-562, TYR-752, and GLY-741, it showed the lowest affinity of -9.2 kcal/mol (**Figure 4F**). When selumetinib bond with TYR-547, SER-630, TYR-662, and ARG-125, the lowest affinity of -7.9 kcal/mol was indicated (**Figure 4G**). All the affinity, root mean square deviation (RMSD) values and active site residues of each binding mode were listed in **Supplementary Table 2**.

To evaluate the dynamic stability of the docked complexes, several key metrics were analyzed from the MD simulation trajectories. The RMSD of the simulation systems was first examined. As shown in **Figure 4H**, all four complex systems reached equilibrium after 20–30 ns, with their RMSD values fluctuating within a stable range of 2-4 Å, indicating that the overall structures were well-maintained. This stability was further corroborated by the Rg, which reflects the structures’ compactness. The Rg​ values for all complexes remained stable with only slight fluctuations, suggesting that no significant unfolding or expansion occurred during the simulations (**Figure 4I**). Furthermore, the impact of ligand binding on the protein’s surface and flexibility was assessed. The SASA of the complexes showed no significant changes, implying that ligand binding induced only minor perturbations to the overall protein structure (**Figure 4J**). Concurrently, the RMSF analysis revealed that the flexibility of most amino acid residues was low (predominantly below 3 Å), confirming the high stability of the complexes (**Figure 4K**). Finally, all four ligands were found to maintain consistent hydrogen bond interactions with DPP4 throughout the simulation, highlighting their favorable engagement with the target (**Supplementary Figure 10C**). In summary, the combination of stable RMSD and Rg​ values, low residue flexibility, and persistent hydrogen bonding collectively provides strong evidence that Saracatinib, Selumetinib, Dasatinib, and Midostaurin all form stable and favorable complexes with the DPP4 target protein.

### 3.7 Single-cell sequencing analysis

To investigate the cellular distribution of DPP4 within the tumor microenvironment (TME), we performed single-cell sequencing analysis. Initially, we explored the TISCH2 database and analyzed datasets GSE137829, GSE143791, and GSE172301, which revealed that DPP4 was predominantly expressed in epithelial cells and CD8+ T cells (**Supplementary Figure 11**). Subsequently, we examined the IMMUcan SingleCell RNA-seq Database, which demonstrated that DPP4 is widely expressed across various immune and stromal cell types in pan-cancer contexts, with particularly high expression levels observed in T cells (**Supplementary Figure 12**).

**Figure 5:**
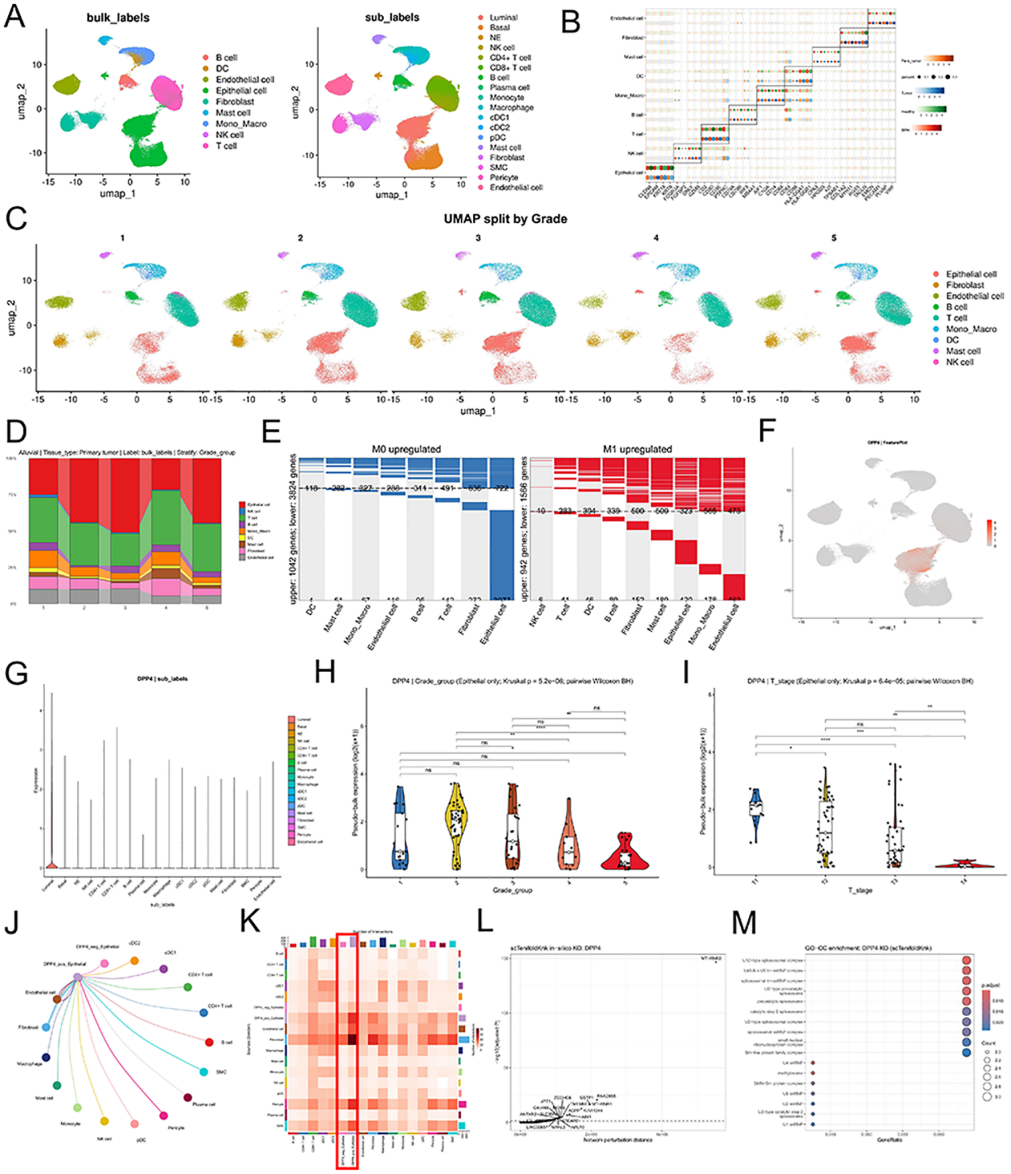
A single-cell transcriptomic atlas of DPP4 in prostate cancer. **(A)** UMAP dimension reduction plot exhibiting 386,664 single-cell transcriptomes across nine major cell lineages (B cell, DC, endothelial cell, epithelial cell, fibroblast, mast cell, mono_macro, NK cell, and T cell) and 18 minor subtypes (luminal, basal, NE, NK cell, CD4+ T cell, CD8+ T cell, B cell, plasma cell, monocyte, macrophage, cDC1, cDC2, pDC, mast cell, fibroblast, SMC, pericyte, and endothelial cell). **(B)** Bubble plots depicting the feature expression of different marker genes in nine major cell subtypes. **(C)** UMAP dimension reduction plots by Grade1-5. **(D)** Bar plot demonstrating that the proportion of epithelial cells varied greatly in different grades of prostate cancer. **(E)** Stacked bar plots highlighting the enrichment of upregulated DEGs in epithelial cells within non-metastatic prostate cancer. **(F, G)** DPP4 expression was exclusively expressed in luminal cells. **(H)** Violin plots comparing DPP4 expression levels across ISUP grades, showing significantly higher expression in low-grade groups (p < 0.001). **(I)** Violin plots showing DPP4 expression across clinical T stages, indicating a significant downregulation in advanced stages (p < 0.001). **(J)** The interaction network illustrating the cellular communications of DPP4+ epithelial cells. **(K)** Heatmap summarizing the total interaction numbers, highlighting that DPP4+ epithelial cells exhibit significant communication with fibroblasts. **(L)** Volcano plot showing genes significantly perturbed by virtual DPP4 knockout in epithelial cells. **(M)** Functional enrichment analysis of the significantly perturbed genes following virtual KO of DPP4. UMAP, Uniform Manifold Approximation and Projection; AJCC, American Joint Committee on Cancer; DEG, Differential Expressed Genes; KO, Knockout.

To further elucidate the cellular distribution and potential functions of DPP4 within the prostate cancer microenvironment, we integrated eight datasets (GSE141445, GSE185344, GSE137829, GSE150692, GSE172301, GSE172316, GSE176031, GSE181294) and constructed a comprehensive single-cell transcriptomic atlas utilizing 386,664 high-quality single-cell transcriptomes. Unsupervised clustering and UMAP dimension reduction identified nine major cell lineages, including epithelial cells, T cells, B cells, and fibroblasts, which were further refined into 18 minor subpopulations (**Figure 5A**). The marker genes were shown in **Figure 5B**, and UMAP dimension reduction plots by Grades were shown in **Figure 5C**. We observed that the proportion of epithelial cells exhibited significant changes across different cancer grades (**Figure 5D**). Within the epithelial compartment of non-metastatic PCa, a dominant number of upregulated DEGs were identified, suggesting active transcriptional remodeling in these cells (**Figure 5E**). The expression of DPP4 was found to be highly cell-type specific, being almost exclusively restricted to the luminal cell subpopulation (**Figures 5F, G**).

Correlation analysis with clinical parameters revealed that DPP4 expression is closely linked to PCa progression. Specifically, DPP4 levels were significantly higher in patients with lower grades and earlier pathological T stages (*p* < 0.001), with a progressive downregulation observed in more advanced and aggressive stages of the disease (**Figures 5I, H**). Besides, to explore how DPP4+ epithelial cells influence the tumor microenvironment, we performed intercellular communication analysis. The interaction network revealed complex crosstalk between DPP4+ epithelial cells and other types of cells (**Figure 5J**). Interestingly, the interaction heatmap highlighted that DPP4+ epithelial cells exhibited the most intensive communication with fibroblasts, suggesting that the DPP4-mediated signaling axis may play a critical role in modulating the tumor-stroma interface in the prostate cancer niche (**Figure 5K**).

### 3.8 Virtual knockout analysis

To further investigate the intracellular regulatory network governed by DPP4 and its potential downstream impact on PCa progression, we performed a virtual knockout (KO) analysis specifically in the epithelial cell population using the scTenifoldKnk framework. The robustness of the virtual KO model was validated by assessing the number of significantly perturbed genes across various False Discovery Rate (FDR) thresholds (**Supplementary Figure 13A**). The global transcriptomic shifts following DPP4 loss were visualized via a volcano plot, identifying a distinct set of genes with significant perturbation levels (**Figure 5L**). Top 30 candidates showing marked sensitivity to DPP4 depletion was shown with bar plot (**Supplementary Figure 13B**). To elucidate the functional architecture of these perturbed genes, we constructed a PPI network (**Supplementary Figure 13C**). Intriguingly, functional enrichment analysis of these significantly perturbed genes provided a deeper mechanistic perspective on the consequences of DPP4 loss. The analysis revealed a striking enrichment in components of the spliceosomal tri-snRNP complex, U2-type precatalytic spliceosome, and methylosome assembly (**Figure 5M**).

### 3.9 TIDE analysis

To further assess the relationship between DPP4 expression and immunotherapy response, we conducted an analysis using the TIDE database. The results indicated that higher DPP4 expression was associated with an improved response to immune checkpoint blockade therapy, as shown by several key findings: increased response rates to immunotherapy (**Supplementary Figure 14A**), reduced CTL infiltration (**Supplementary Figure 14B**), lower TIDE scores (**Supplementary Figure 14C**), and decreased tumor T cell dysfunction potential (**Supplementary Figure 14D**) and tumor T cell exclusion potential (**Supplementary Figure 14E**). These results suggest that elevated DPP4 expression correlates with a better response to immunotherapy.

### 3.10 Baseline information of the validation clinical cohort

**Figure 6:**
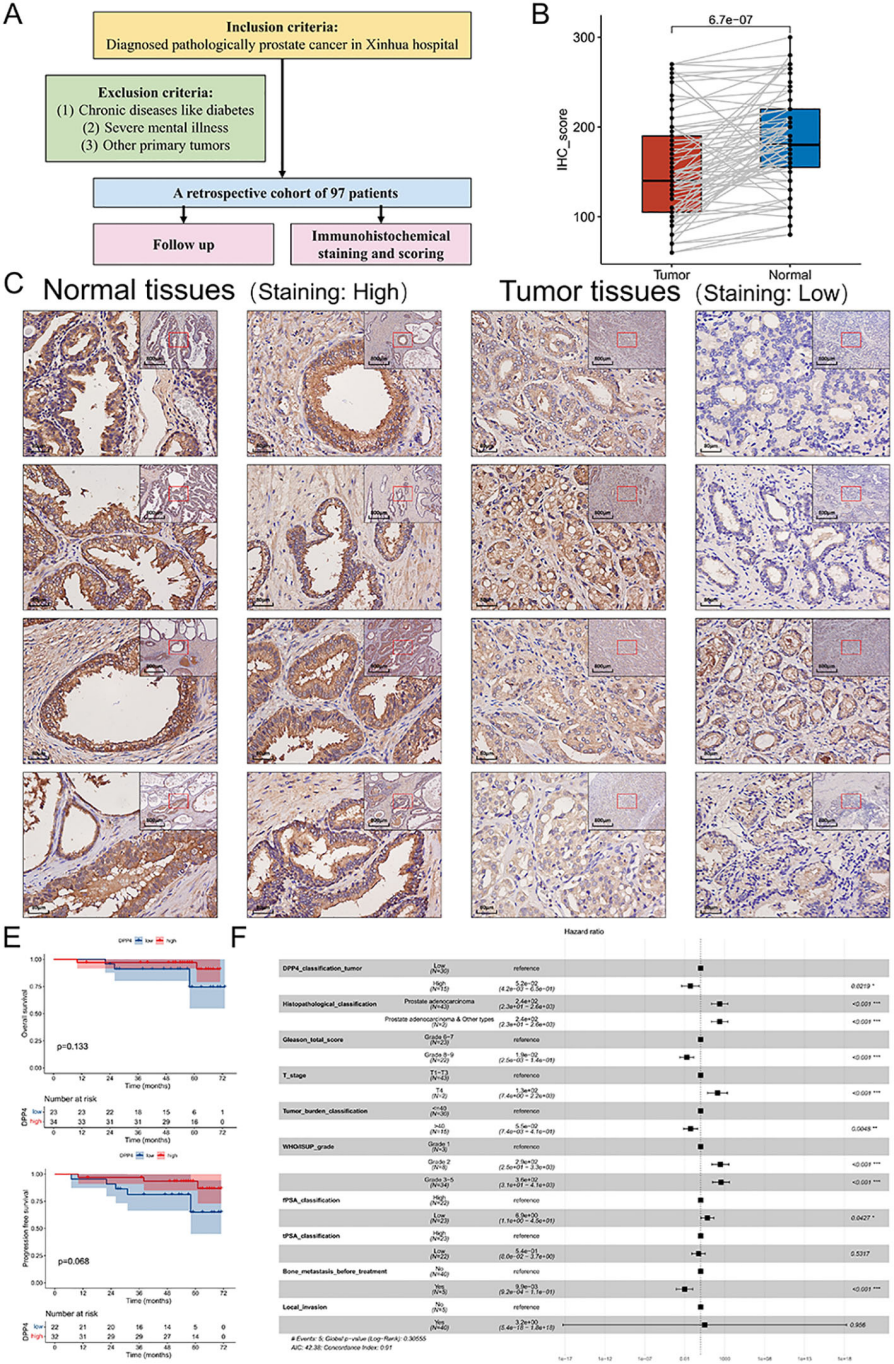
Higher DPP4 expression was correlated with better prognosis in prostate cancer. **(A)** The inclusion and exclusion criteria of the cohort. **(B)** IHC scores revealed that normal tissue exhibited significantly higher DPP4 expression compared to tumor tissues (*p* < 0.001). **(C)** Representative IHC images of both prostate cancer and normal tissues from the cohort demonstrated this difference visually. **(D)** The Chi square test showed that DPP4 expression was associated with WHO/ISUP grade (*p* = 0.03). **(E)** The K-M survival curve indicated that higher DPP4 expression was not significantly correlated with OS and PFS (*p* > 0.05). However, DPP4 expression tended to be a protective factor. **(F)** Multivariate Cox regression analysis indicated that high DPP4 expression was an independent protective factor for OS in prostate cancer patients (HR = 0.052, 95%CI = 0.0041 - 0.65, *p* = 0.02). IHC, Immunohistochemical; OS, Overall Survival; PFS, Progression-Free Survival.

Through our analysis of DPP4 in pan-cancer, it was found that DPP4 was significantly correlated with prognosis in PRAD. In order to validate its oncogenic effect in PRAD, we conducted a retrospective clinical cohort. A total of 97 prostate cancer patients from Xinhua Hospital were included in this retrospective cohort study. The inclusion and exclusion criteria are depicted in **Figure 6A**, and the demographic and clinical characteristics are presented in **Table 1** and **Supplementary Figure 15A**. Histopathological classification revealed that the majority of patients (93.81%) were diagnosed with prostate adenocarcinoma, while smaller percentages were diagnosed with prostate adenocarcinoma & prostate ductal adenocarcinoma (2.06%), and prostate adenocarcinoma & prostate mucinous adenocarcinoma (1.03%). Clinically, 59.79% of patients were alive, and 52.58% had not experienced disease progression.

**Table 1:** Demographic characteristics of 97 patients.

Variables	Number (%)	Mean ± SD	Median (range)	P-value
**DPP4 tumor**		153.63 ± 56.76	142.5 (60-270)	
**DPP4 classification tumor**				
Low (IHC score of DPP4 <= 135)	53 (54.64)			
High (IHC score of DPP4 > 135)	31 (31.96)			
Unknown	13 (13.40)			
**DPP4 normal**		184.79 ± 53.49	180 (80-300)	
**DPP4 classification normal**				< 0.05*
Low (IHC score of DPP4 <= 135)	25 (25.77)			
High (IHC score of DPP4 > 135)	48 (49.78)			
Unknown	24 (24.74)			
**Age**		70.56 ± 7.64	70 (46-86)	
**Age category**				
46-80	88 (90.72)			
81-86	9 (9.28)			
**Histopathological classification**				
Prostate adenocarcinoma	91 (93.81)			
Prostate adenocarcinoma & Prostate ductal adenocarcinoma	2 (2.06)			
Prostate adenocarcinoma & Prostate mucinous adenocarcinoma	1 (1.03)			
**Gleason grade**				
3 + 3 = 6	7 (7.22)			
3 + 4 = 7	15 (15.46)			
3 + 5 = 8	7 (7.22)			
4 + 3 = 7	23 (23.71)			
4 + 4 = 8	9 (9.28)			
4 + 5 = 9	24 (24.74)			
5 + 4 = 9	1 (1.03)			
Unknown	11 (11.34)			
**Gleason total score**				< 0.05*
Grade 6	7 (7.22)			
Grade 7	38 (39.18)			
Grade 8	16 (16.49)			
Grade 9	25 (25.77)			
Unknown	11 (11.34)			
**WHO/ISUP grade**				< 0.05*
Grade 1	7 (7.22)			
Grade 2	15 (15.46)			
Grade 3	23 (23.71)			
Grade 4	16 (16.49)			
Grade 5	25 (25.77)			
Unknown	11 (11.34)			
**Tumor burden**		40.19 ± 24.43	40 (1-95)	
**Tumor burden classification**				
<=40	53 (54.64)			
>40	32 (32.99)			
Unknown	12 (12.37)			
**Local invasion**				
Yes	75 (77.32)			
No	19 (19.59)			
Unknown	3 (3.09)			
**T stage**				
T1	3 (3.09)			
T2	28 (28.87)			
T3	45 (46.39)			
T4	7 (7.22)			
Unknown	14 (14.43)			
**Detailed T stage**				
T1a	1 (1.03)			
T1c	1 (1.03)			
T2a	2 (2.06)			
T2b	1 (1.03)			
T2c	25 (25.77)			
T3a	26 (26.80)			
T3b	18 (18.56)			
T4	7 (7.22)			
Unknown	16 (16.49)			
**N stage**				
N0	39 (40.21)			
N1	13 (13.40)			
Nx	19 (19.59)			
Unknown	26 (26.80)			
**M stage**				
M0	1 (1.03)			
Mx	69 (71.13)			
Unknown	27 (27.84)			
**Radiotherapy**				
Yes	6 (6.19)			
No	91 (93.81)			
**Chemotherapy**				
Yes	8 (8.25)			
No	89 (91.75)			
**Endocrinotherapy**				
Yes	29 (29.90)			
No	68 (70.10)			
**Serum alkaline phosphatase (UL)**		74.20 ± 24.45	70 (40.6-188.4)	
**Serum alkaline phosphatase classification**				
High	41 (42.27)			
Low	42 (43.30)			
Unknown	14 (14.43)			
**fPSA (ngml)**		6.73 ± 48.53	0.23 (0.00-470.64)	
**fPSA classification**				
High	48 (49.48)			
Low	49 (50.52)			
**tPSA (ngml)**		41.45 ± 162.46	13.41 (0.00-1573.00)	
**tPSA classification**				
High	48 (49.48)			
Low	49 (50.52)			
**fPSA/tPSA**		3.47 ± 4.85	1.86 (0.01-29.92)	
**fPSA/tPSA classification**				
High	47 (48.45)			
Low	50 (51.55)			
**Bone metastasis before treatment**				
Yes	18 (18.56)			
No	79 (81.44)			
**Biochemical recurrence after treatment**				
Yes	6 (6.19)			
No	53 (54.64)			
Unknown	38 (39.18)			
**Progression after treatment**				
Yes	13 (13.40)			
No	51 (52.58)			
Unknown	33 (34.02)			
**Progression free survival**		51.27 ± 18.06	57.5 (7-73)	
**OS censor**				
Alive	58 (59.79)			
Dead	10 (10.31)			
Unknown	29 (29.90)			
**Overall survival**		53.02 ± 16.40	58 (10-73)	

IHC, Immunohistochemical; PSA, Prostate Specific Antigen; fPSA, free PSA; tPSA, total PSA. *p < 0.05, **p < 0.01, ***p < 0.001.

Bold typeface denotes the names of primary variables, including both categorical and continuous parameters.

### 3.11 DPP4 expression and clinical relevance in prostate cancer

IHC staining was performed on tumor and normal tissues from the 97 patients. DPP4 was significantly downregulated in prostate cancer tumor tissues (*p* < 0.001), with a mean IHC score of 153.63 in tumor tissues compared to 184.79 in normal tissues (**Figure 6B**). Representative IHC images of both tumor and normal tissues from the cohort are shown in **Figure 6C**.

Using the “*surv_cutpoint*” method, the cut-off value for the IHC score of DPP4 in tumor tissues was determined to be 135 for both OS and PFS (**Supplementary Figure 15B** and **Supplementary Figure 15C**). K-M survival analysis demonstrated that DPP4 expression in tumor tissues was not significantly associated with or PFS (*p* > 0.05), but it showed a protective trend (**Figure 6E**). However, multivariate Cox regression analysis indicated that high DPP4 expression was a protective factor for OS in prostate cancer patients (HR = 0.052, 95%CI = 0.0041 - 0.65, *p* = 0.02) (**Figure 6F**).

### 3.12 Drug sensitivity in prostate cancer

**Figure 7:**
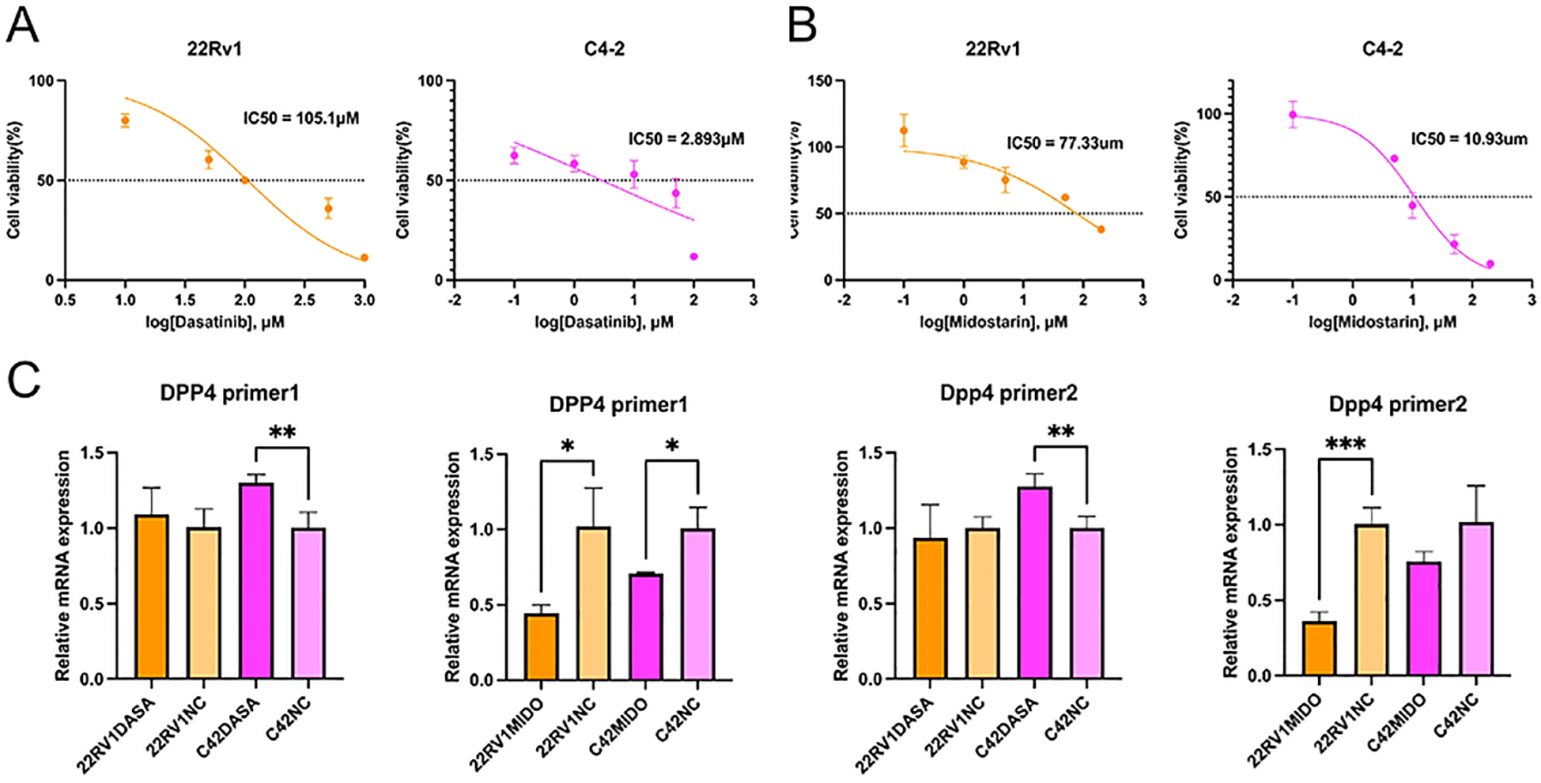
Dasatinib and midostaurin regulated DPP4 expression. **(A)** IC50 of 22Rv1 and C4–2 treated with dasatinib tested by CCK-8 assays. **(B)** IC50 of 22Rv1 and C4–2 treated with midostarin tested by CCK-8 assays. **(C)** Dasatinib treatment significantly increased DPP4 expression in C4–2 cells (*p* = 0.0042, primer 1; *p* = 0.0029, primer 2). In contrast, midostaurin treatment reduced DPP4 expression in both cell lines (C4-2: *p* = 0.0218, primer 1; 22Rv1: *p* = 0.0172, primer 1; *p* = 0.0002, primer 2). IC50, Half maximal inhibitory concentration; CCK-8, Cell Counting Kit-8.

The half-maximal inhibitory concentration (IC_50_) of dasatinib was 105.1 µM in 22Rv1 cells and 2.893 µM in C4–2 cells (**Figure 7A**). For midostaurin, the IC_50_ was 77.33 µM in 22Rv1 cells and 10.93 µM in C4–2 cells (**Figure 7B**). qPCR analysis revealed that dasatinib treatment significantly increased DPP4 expression in C4–2 cells (*p* = 0.0042, primer 1; *p* = 0.0029, primer 2). In contrast, midostaurin treatment led to a significant reduction of DPP4 expression in both cell lines (C4-2: *p* = 0.0218, primer 1; 22Rv1: *p* = 0.0172, primer 1; *p* = 0.0002, primer 2) (**Figure 7C**).

## 4 Discussion

Dipeptides play a key role in tumor (55). Dipeptidyl peptidase 4 (DPP4), a transmembrane glycoprotein, is known for its role in degrading circulating glucagon-like peptide-1 (GLP-1) and glucose-dependent insulinotropic peptide (GIP) (56). DPP4 inhibitors, commonly referred to as gliptins, are oral antidiabetic drugs (OADs) used in treating type 2 diabetes mellitus (T2DM) by extending the half-life of native incretins (57, 58). Additionally, DPP4 has various functions in the immune system, such as cleaving cytokines, chemokines, and peptide hormones involved in immune regulation (59), as well as modulating lymphocyte functions (2). However, there are few studies researching its role in tumor.

In this research, we found that DPP4 was significantly downregulated in tumor tissues, and higher DPP4 expression was associated with better OS and PFS. Its potential tumor-suppressing property was highlighted in our study, which was in line with previous researches (11, 12, 60). What’s more, the relationship between DPP4 and metastasis is worth exploring in PCa, since it seems to be linked to the metastatic cascade due to its peptidase activity (61, 62). The exact mechanism of DPP4 act in PCa remains unclear and needs further investigation.

Interestingly, a previous pan-cancer study focused on DPP4 in COVID-19-affected patients, finding that increased DPP4 expression in specific cancer patients might account for the high susceptibility to SARS-CoV-2 infection and the induction of cytokine storms (63). Compared to the 2021 study, which primarily explored DPP4 expression in the context of SARS-CoV-2 susceptibility, our study takes a more comprehensive approach by analyzing DPP4’s prognostic significance, immune associations, and therapeutic relevance across multiple cancer types. Importantly, we also provide clinical validation in a cohort of 97 prostate cancer patients, adding translational value that was absent from the earlier research.

### 4.1 DPP4 is a potential pan-cancer biomarker

Previous research has investigated the correlation between DPP4 and tumor development, as well as patient prognosis, yielding controversial results. Barreira da Silva et al. discovered that DPP4 inhibition boosts anti-tumor responses in melanoma (5). Hollande et al. reported that DPP4 inhibition promotes anti-tumor responses by facilitating eosinophil migration into HCC and breast carcinoma (6). Conversely, Russo et al. found that inhibiting DPP4 increases the growth of prostate cancer (PCa) xenografts following castration (12). Additionally, Xie et al. suggested that DPP4 is essential for ferroptosis, a form of regulated cell death that could help selectively eliminate tumor cells in CRC (9).

In our pan-cancer study, we observed significant variations in DPP4 expression across different cancer types. DPP4 expression was notably downregulated in BRCA, CESC, CHOL, COAD, KICH, LUSC, PCPG, READ, and UCEC, while it was upregulated in GBM, KIRC, KIRP, LIHC, LUAD, STAD, and THCA. Regarding clinical prognosis, we found that DPP4 expression was negatively correlated with OS in BLCA, PRAD, and LGG, but positively correlated in KIRC, KIRP, MESO, and THYM. For DFS, DPP4 was negatively associated with DFS in LUAD, yet positively correlated in PRAD. When examining DSS, DPP4 showed a negative correlation with DSS in LGG and LUSC, but a positive correlation in KIRC, KIRP, MESO, and SKCM. As for PFS, DPP4 was negatively associated with PFS in LGG and LUSC, while it was positively correlated in KIRC, MESO, and PRAD. Additionally, DPP4 expression was positively correlated with the clinical stages of KIRC and TGCT. In our clinical validation study, we established a retrospective cohort and observed that DPP4 expression was significantly downregulated in the tumor tissues of prostate cancer patients. Additionally, patients with high DPP4 expression had notably longer OS. These findings highlight the potential of DPP4 as a biomarker for pan-cancer, especially prostate cancer, providing valuable insights for the development of personalized treatment strategies.

### 4.2 DPP4 actively participates in the TME

The TME is composed of a diverse array of immune cells, cancer-associated fibroblasts, endothelial cells, pericytes, and other cell types, which collectively play a crucial role in tumor growth, metastasis, and treatment response (64, 65). Recent studies have used scRNA-seq and bioinformatics to reveal that CD8+ T-cell exhaustion-related lncRNAs, acting through ceRNA networks, serve as key prognostic markers and immune regulators in cancer (66). Previous studies have shown that DPP4 has various functions in the immune system. For instance, the DPP4-ADA-adenosine pathway is considered essential for T-cell activation (67). In addition to T lymphocytes, DPP4 has been found to play a role in B cells (68), natural killer (NK) cells (10), dendritic cells (DCs) (69), and macrophages (70).

In this study, we discovered that DPP4 expression was positively correlated with immune scores in BLCA, BRCA, COAD, DLBC, GBM, HNSC, LAML, LGG, LUAD, LUSC, OV, PCPG, SARC, SKCM, THCA, THYM, UCEC, and UVM, while it was negatively correlated in KIRC and LIHC. For immune gene co-expression, the majority of immune genes showed a positive correlation with DPP4 expression in BLCA, BRCA, LGG, SKCM, and THCA.

Mechanistically, our PPI network analysis revealed a critical interaction between DPP4 and the CXCL12/CXCR4 signaling axis. DPP4, acting as a cell-surface protease, is known to facilitate the N-terminal cleavage of the chemokine CXCL12 (SDF-1α), which subsequently abolishes its affinity for the CXCR4 receptor (60). In the context of prostate cancer, the CXCL12/CXCR4 axis is a well-documented driver of epithelial-to-mesenchymal transition (EMT) and bone metastasis (60, 71). Therefore, the significant downregulation of DPP4 observed in our PCa cohort likely leads to an accumulation of active CXCL12 in the tumor microenvironment, thereby amplifying oncogenic signaling. Furthermore, recent evidence has identified DPP4 as an androgen receptor (AR)-regulated tumor suppressor, whose loss of expression accelerates the progression to castration-resistant prostate cancer (CRPC) (12). This aligns closely with our clinical observations. We further observed that dasatinib treatment upregulated DPP4 expression in C4–2 cells, suggesting that the therapeutic efficacy of this inhibitor may be partially attributed to the restoration of DPP4-mediated chemokine regulation, thereby suppressing the malignant phenotypes driven by the CXCL12 axis.

DPP4 expression showed a strong association with CD4+ T cells, CD8+ T cells, and regulatory T cells (Tregs). Moreover, the TIMER 2.0 database revealed that DPP4 was positively correlated with cancer-associated fibroblasts (CAF), macrophages, endothelial cells, and neutrophils in nearly all tumor types. CAFs play a crucial role in tumor development by promoting cancer cell proliferation, therapy resistance, and immune exclusion (72). They achieve this by releasing cytokines and growth factors, creating extracellular matrix (ECM) structures, and reprogramming the tumor microenvironment, which contributes to chemotherapy resistance and tumor progression (73). Macrophages, as a universal component of the TME, engage in complex interactions with cancer cells, stroma, and immune cells. They have the ability to kill tumor cells, mediate antibody-dependent cellular cytotoxicity and phagocytosis, induce vascular damage and tumor necrosis, and activate both innate and adaptive immune responses against tumors (74). Neutrophils are known to promote tumor neovascularization (75). Activated neutrophils can secrete various pro-angiogenic factors that directly influence tumor angiogenesis (76). Endothelial cells are critical for tumor-associated angiogenesis and are a major source of CAFs (77). These cells can undergo endothelial-to-mesenchymal transition, enhancing the stromal fibroblast microenvironment and reshaping the vascular system to support tumor cell invasion and metastasis (78). The correlation between DPP4 expression and the infiltration of CAFs, macrophages, endothelial cells, and neutrophils may explain the better prognosis observed in patients with upregulated DPP4.

As DPP4 may be involved in the TME of PCa, its relationship with the AR warrants further exploration. In PCa, DPP4 has been shown to interact with various growth factors, such as fibroblast growth factor 2 (FGF2) and CXC chemokine ligand 12 (CXCL12), both of which play key roles in tumor progression, immune modulation, and metastasis (11, 60). For instance, FGF2 can influence the biological behavior of neoplastic cells by regulating AR expression, leading to downregulation of AR protein levels (79, 80). Additionally, activation of the CXCL12/CXCR4 axis promotes AR-dependent gene transcription, cellular proliferation, and PSA secretion (81). DPP4 may interact with these growth factors, potentially altering downstream AR expression. In conclusion, DPP4 expression is closely associated with the composition of the TME and warrants further investigation.

### 4.3 DPP4 could serve as a promising marker for guiding immunotherapy

Given that TMB and MSI are significant prognostic biomarkers across various tumors and reliable predictors for immunotherapy (82), understanding their correlation with DPP4 expression is crucial. High TMB and MSI levels in certain tumors are associated with better prognoses and greater efficacy of immunotherapy (83).

In our study, DPP4 expression was negatively associated with MSI in DLBC, HNSC, LUSC, PRAD, SKCM, and UCS, while a positive correlation was observed in COAD, ESCA, and KIRC. Additionally, DPP4 expression was negatively correlated with TMB in several cancer types, including THYM, LUSC, CESC, PRAD, BRCA, and LUAD. Conversely, a positive correlation was noted in LAML, SARC, ESCA, KIRP, COAD, UCEC, GBM, LIHC, and OV.

These findings suggest that DPP4 expression levels may influence TMB and MSI across different cancers, thereby affecting immunotherapy responses. Furthermore, cBioPortal and the GSCA database revealed the mutation patterns of DPP4. Since tumors with high levels of somatic mutations due to mismatch-repair deficiencies show increased sensitivity to PD-1 targeted therapy (84), DPP4 may have promising applications in immune checkpoint blockade therapy.

### 4.4 Functional analysis indicates a significant correlation between DPP4 and both immunity and metabolism

To gain deeper insight into the influence of DPP4 on tumor development, we identified enriched signaling pathways using GSEA. We found five pathways to be most significantly correlated: “ascorbate and aldarate metabolism”, “olfactory transduction”, “pentose and glucuronate interconversions”, “porphyrin and chlorophyll metabolism”, and “starch and sucrose metabolism”. “Ascorbate and aldarate metabolism” is a vital carbohydrate metabolic pathway that protects cells from oxidative damage (85), and also plays an important role in eliminating cancer cells. Ascorbate has been found to selectively kill certain cancer cell types (86), a process dependent on hydrogen peroxide formation (87). “Olfactory transduction” is not limited to the olfactory sensory neurons. Olfactory receptors (ORs) are expressed in prostate tissues (88), and their activation has been suggested to contribute to PCa pathogenesis and progression (89). “Pentose and glucuronate interconversions” is crucial for cancer cells as it generates pentose phosphates for the high rate of nucleic acid synthesis in tumors and provides NADPH, necessary for fatty acid synthesis and cell survival under stress conditions (90). In summary, the enrichment of these five signaling pathways suggests that DPP4 is associated with immunity and metabolism in cancer. Further investigation is needed to elucidate the exact mechanisms by which DPP4 functions in pan-cancer contexts.

### 4.5 DPP4 influences drug sensitivity and suggests potential therapeutic strategies in cancer

We also performed drug sensitivity prediction analysis in order to shed light on the therapeutic potential of DPP4. Since higher DPP4 expression was associated with decreased drug sensitivity of most drugs, it implies that DPP4 may involve in mechanisms that lead to greater therapeutic challenges. Few drugs showed higher sensitivity, including perifosine and adavosertib from CellMiner, dasatinib and saracatinib from CTRP, and cetuximab and crizotinib from GDSC. Perifosine, a synthetic oral alkylphosphocholine, induces a wide range of antitumor effects. It can lead to delayed tumor cell proliferation via cell cycle arrest, direct cytotoxicity through apoptosis induction, and antiangiogenesis across various tumor types (91, 92). Adavosertib, a WEE1 inhibitor, disrupts the S-G2 checkpoint function by uncontrolled activation of CDK1/2, resulting in tumor cell death (93). It has shown promising antitumor efficacy in multiple solid tumors (94, 95). Dasatinib and saracatinib, ATP-competitive SRC/SFK inhibitors (96, 97), can inhibit prostate tumor growth and metastasis while also targeting osteoclast activity and bone resorption *in vitro* (98). Cetuximab, a human-murine chimeric anti-EGFR monoclonal antibody (99), is widely used in treating various cancers, including colorectal cancer, head and neck cancer, and lung cancer (100). Crizotinib is the first ALK inhibitor approved for treating late-stage lung cancer, anaplastic large cell lymphoma, and neuroblastoma. It is also the first drug specifically targeting non-small cell lung carcinoma (NSCLC) patients (101). While earlier research has not directly linked DPP4 with these drugs, our findings provide valuable insights into DPP4’s potential pharmaceutical applications. To advance our investigation, we conducted molecular docking analysis to identify the potential binding site between DPP4 and dasatinib, midostaurin, saracatinib and selumetinib, as well as performed MD simulation to validate the binding stability. This suggests that DPP4 could serve as a biomarker for predicting the response of prostate cancer patients to these drugs. Further molecular-level experiments are needed to validate this connection. Since drug sensitivity is positively correlated with DPP4 expression, identifying high DPP4 levels in tumors could help guide the use of these drugs for treatment.

For *in vitro* validation, dasatinib markedly increased DPP4 expression in C4–2 cells, whereas midostaurin consistently suppressed DPP4 expression in both 22Rv1 and C4–2 cells. These findings suggest that DPP4 is not only responsive to kinase inhibition but may also participate in distinct signaling pathways influenced by these agents. Given the established roles of DPP4 in tumor progression, immune modulation, and drug resistance, the observed regulation highlights its potential as a downstream effector or biomarker for therapeutic response in prostate cancer. Moreover, the divergent effects of dasatinib and midostaurin imply that targeting DPP4 may yield context-dependent outcomes, underscoring the need for further mechanistic studies to clarify its functional contribution and therapeutic value.

### 4.6 Limitations

Our study has some limitations. The expression data for DPP4 and clinical data across different tumor types were gained from TCGA database, whose predominant population is Caucasian and samples are derived from medical institutions in the United States, potentially introducing geographic and ethnic biases. Furthermore, our pan-cancer analysis was based solely on bioinformatics technology, which does not provide detailed insights into the underlying molecular mechanisms. Consequently, subsequent studies incorporating experimental validation of downstream signaling pathways are necessary. Further research is needed to fully understand the mechanisms regulating DPP4 expression, including exploring the relationship between DPP4 and miRNAs, as well as miRNAs and lncRNAs. Moreover, the levels of dipeptides generated by DPP4 warrant further investigation, and the potential effects of DPP4 inhibitors such as gliptins remain to be thoroughly validated. Finally, our docking experiments and MD simulations lack direct biochemical validation. Despite these limitations, we hope our study can serve as a foundation for future research into DPP4 and its potential as a target for anti-cancer therapies. Further investigations are needed to fully elucidate the mechanisms of DPP4.

## 5 Conclusion

In this study, we investigated the role of DPP4 in a pan-cancer context and its impact on prostate cancer progression using a large retrospective cohort. Our results indicate that DPP4 significantly influences the TME and may serve as a potential marker for immunotherapy. Additionally, DPP4 shows associations with immune response, metabolism, and drug sensitivity. Thus, DPP4 has the potential to be a biomarker for predicting immunotherapy efficacy and guiding personalized cancer treatment. Further research is necessary to elucidate the molecular mechanisms underlying DPP4’s functions.

## Data Availability

The original contributions presented in the study are included in the article/**Supplementary Material**. Further inquiries can be directed to the corresponding authors.
