# Supplementary material for: Integrative pan-cancer analysis of dipeptidyl peptidase 4 with clinical and in vitro validation in prostate cancer
Source: Front Immunol. 2026 Mar 12;17:1616889. doi: 10.3389/fimmu.2026.1616889 (PMC13017899; doi:10.3389/fimmu.2026.1616889)
Supplement: Supplementary file 1 [file DataSheet1.pdf]

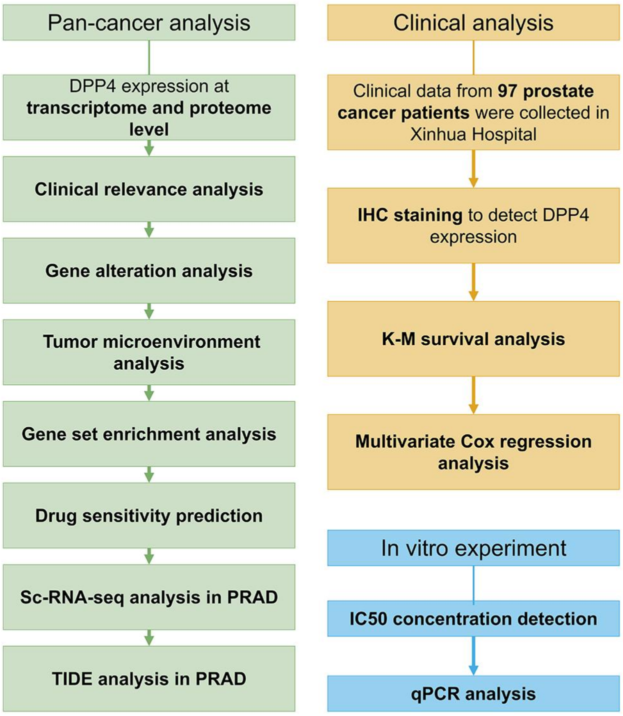

Figure S1. The flowchart of the study. *IHC, immunohistochemical; K-M, Kaplan-Meier.*

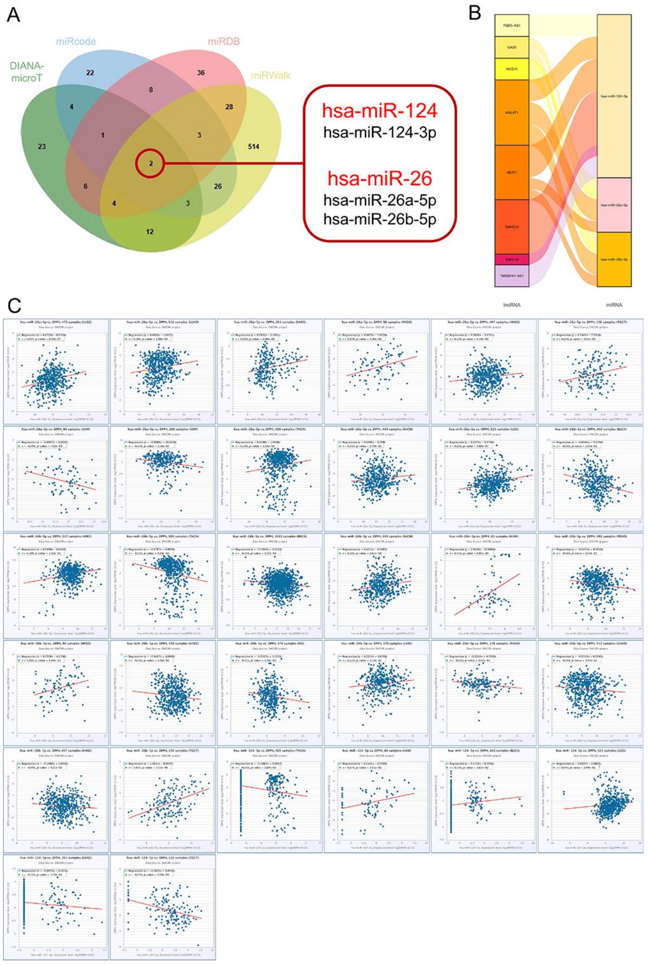

Figure S2. The construction of DPP4 ceRNA network. (**A**) The intersection of four miRNA databases

(miRcode, Diana-microT, miRWalk and miRDB) identified two miRNAs, including hsa-miR-124 and has-miR-26. (**B**) The interaction between miRNA and lncRNA. The three miRNAs included hsa-miR-124-3p, hsa-miR-26a-5p, and hsa-miR-26b-5p. The eight lncRNAs included FGD5-AS1, GAS5, HCG11, MALAT1, NEAT1, SNHG14, SNHG16 and TMEM147-AS1. (**C**) The expression level of miRNAs with DPP4 expression in pan-cancer.

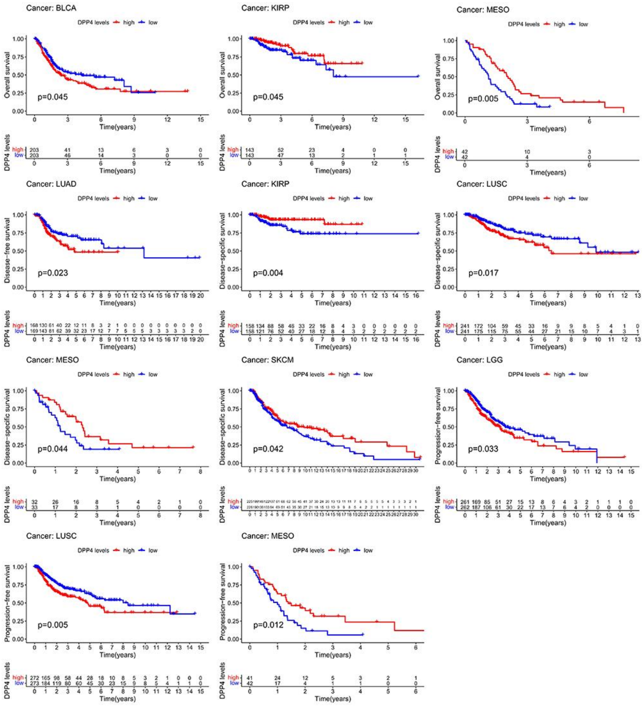

Figure S3. Survival analysis between low- and high-DPP4 expression groups in OS, DFS, DSS and PFS. For OS, DPP4 was negatively correlated with OS in BLCA ($p = 0.045$), while it was positively correlated in KIRP ($p = 0.045$) and MESO ($p = 0.005$). For DFS, DPP4 was negatively correlated in LUAD ($p = 0.023$), but positively correlated in PRAD ($p = 0.013$). For DSS, DPP4 was negatively correlated in LUSC ($p = 0.017$), but positive correlated in KIRP ($p = 0.004$), MESO ($p = 0.044$) and SKCM ($p = 0.042$). For PFS, DPP4 was negatively correlated in LGG ($p = 0.033$), LUSC ($p = 0.005$), but positively correlated in MESO ($p = 0.012$).

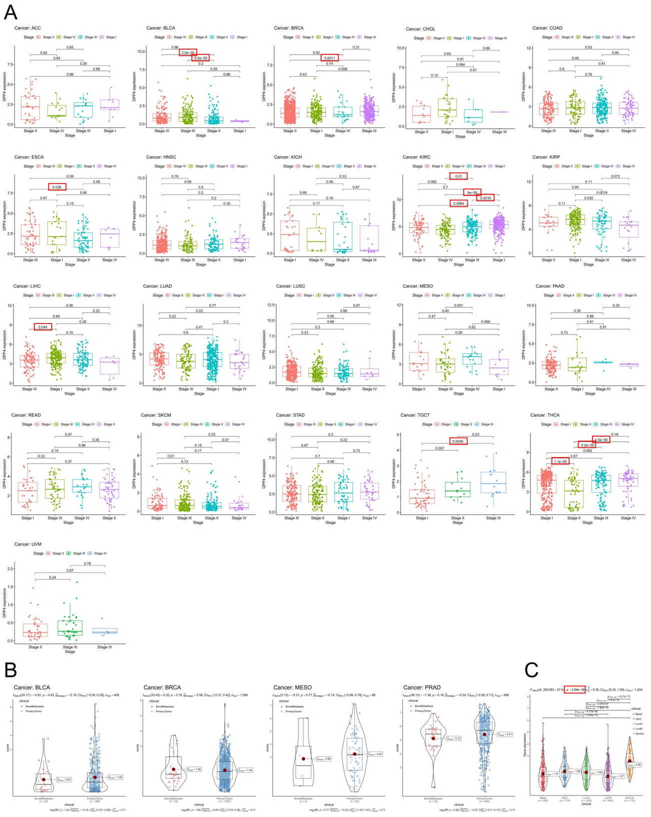

Figure S4. Clinical relevance analysis across clinical stages, bone metastasis, and molecular subtypes. (**A**) Clinical stage analysis. DPP4 exhibited a positive correlation with clinical stage in KIRC and TGCT. (**B**) Bone metastasis analysis. There was no significant difference in DPP4 expression between primary tumors and metastatic tumors in BLCA, BRCA, MESO, or PRAD. (**C**) Molecular subtype analysis. DPP4 expression was notably associated with molecular subtypes in BRCA. Specifically, the normal Luminal B subtype exhibited the lowest DPP4 expression.

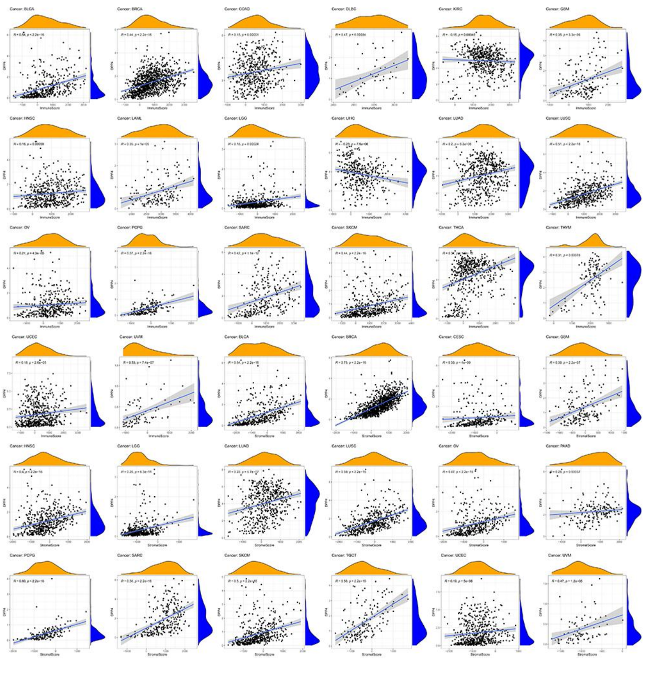

Figure S5. For immune score, the expression of DPP4 was positively correlated in BLCA (R = 0.64, $p < 0.001$), BRCA (R = 0.44, $p < 0.001$), COAD (R = 0.15, $p < 0.001$), DLBC (R = 0.47, $p < 0.001$),

GBM (R = 0.35, $p < 0.001$), HNSC (R = 0.16, $p < 0.001$), LAML (R = 0.35, $p < 0.001$), LGG (R = 0.16, $p < 0.001$), LUAD (R = 0.2, $p < 0.001$), LUSC (R = 0.51, $p < 0.001$), OV (R = 0.21, $p < 0.001$), PCPG (R = 0.57, $p < 0.001$), SARC (R = 0.42, $p < 0.001$) , SKCM (R = 0.44, $p < 0.001$), THCA (R = 0.31, $p < 0.001$), THYM (R = 0.31, $p < 0.001$), UCEC (R = 0.18, $p < 0.001$), and UVM (R = 0.53, $p < 0.001$), while it was negatively correlated in and KIRC (R = -0.15, $p < 0.001$) and LIHC (R = -0.23, $p < 0.001$). For stromal scores, the expression of DPP4 was positively correlated in BLCA (R = 0.64, $p < 0.001$), BRCA (R = 0.73, $p < 0.001$), CESC (R = 0.33, $p < 0.001$), GBM (R = 0.39, $p < 0.001$), HNSC (R = 0.4, $p < 0.001$), LGG (R = 0.28, $p < 0.001$), LUAD (R = 0.22, $p < 0.001$), LUSC (R = 0.59, $p < 0.001$), OV (R = 0.47, $p < 0.001$), PAAD (R = 0.26, $p < 0.001$), PCPG (R = 0.69, $p < 0.001$), SARC (R = 0.56, $p < 0.001$), SKCM (R = 0.5, $p < 0.001$), TGCT (R = 0.56, $p < 0.001$), UCEC (R = 0.19, $p < 0.001$), and UVM (R = 0.47, $p < 0.001$). *MSI, Microsatellite Instability; TMB, Tumor Mutation Burden; TME, Tumor Microenvironment.* *$p < 0.05$, **$p < 0.01$, ***$p < 0.001$.*

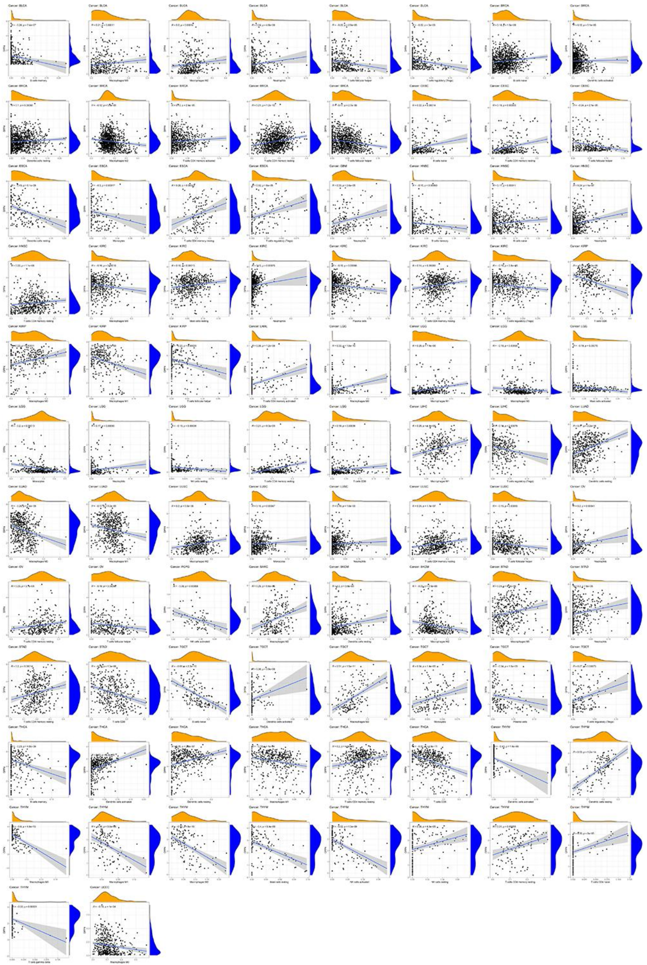

Figure S6. ESTIMATE analysis of correlation between DPP4 expression and immune cell infiltration in pan-cancer. In BLCA, DPP4 expression was negatively correlated with B cells memory, T cells follicular helper, Tregs, while it was positively correlated with macrophages Mo0, macrophages M2, and neutrophils. In BRCA, DPP4 expression was negatively correlated with macrophages M2 and T cells follicular helper, while it was positively correlated with B cells naïve, dendritic cells activated, dendritic cells resting, T cells CD4 memory activated, and T cells CD4 memory resting. In CESC, DPP4 expression was negatively correlated with T cells follicular helper, while it was positively correlated with B cells naïve and T cells CD4 memory resting. In ESCA, DPP4 expression was

negatively correlated with dendritic cells resting and monocytes, while it was positively correlated with T cells CD4 memory resting and Tregs. In GBM, DPP4 was positively correlated with neutrophils. In HNSC, DPP4 expression was negatively correlated with B cells memory, while it was positively correlated with B cells naïve, neutrophils, and T cells CD4 memory resting. In KIRC, DPP4 expression was negatively correlated with macrophages M0, plasma cells, and Tregs, while it was positively correlated with mast cells resting, neutrophils, and T cells CD4 memory resting. In KIRP, DPP4 expression was negatively correlated with T cells CD8, macrophages M1, and T cells follicular helper, while it was positively correlated with macrophages M0. In LAML, DPP4 expression was positively correlated with T cells CD4 memory activated. In LGG, DPP4 expression was negatively correlated with macrophages M2, mast cells activated, monocytes, and NK cells resting, while it was positively correlated with macrophages M0, macrophages M1, neutrophils, T cells CD4 memory resting, and T cells CD8. In LIHC, DPP4 expression was negatively correlated with Tregs, while it was positively correlated with macrophages M1. In LUAD, DPP4 expression was negatively correlated with macrophages M0 and macrophages M1, while it was positively correlated with dendritic cells resting. In LUSC, DPP4 expression was negatively correlated with T cells follicular helper, while it was positively correlated with macrophages M2, monocytes, neutrophils, and T cells CD4 memory resting. In OV, DPP4 expression was negatively correlated with T cells follicular helper, while it was positively correlated with neutrophils and T cells CD4 memory resting. In PCPG, DPP4 expression was negatively correlated with NK cells activated. In SARC, DPP4 expression was positively correlated with macrophages M2. In SKCM, DPP4 expression was negatively correlated with macrophages M2, it was positively correlated with dendritic cells resting. In STAD, DPP4 expression was negatively correlated with T cells CD8, while it was positively correlated with macrophages M0, neutrophils, and T cells CD4 memory resting. In TGCT, DPP4 expression was negatively correlated with B cells naïve and plasma cells, while it was positively correlated with dendritic cells activated, macrophages M2, monocytes, and Tregs. In THCA, DPP4 expression was negatively correlated with B cells memory, macrophages M1, and T cells CD, while it was positively correlated with dendritic cells activated, dendritic cells resting, and T cells CD4 memory resting. In THYM, DPP4 expression was negatively correlated with dendritic cells activated, macrophages M0, macrophages M1, macrophages M2, mast cells resting, NK cells activated, and T cells gamma delta, while it was positively correlated with dendritic cells resting, NK cells resting, T cells CD4 memory resting, and T cells CD4 naïve. In UCEC, DPP4 expression was negatively correlated with macrophages M0. *Tregs, T cells regulatory.*

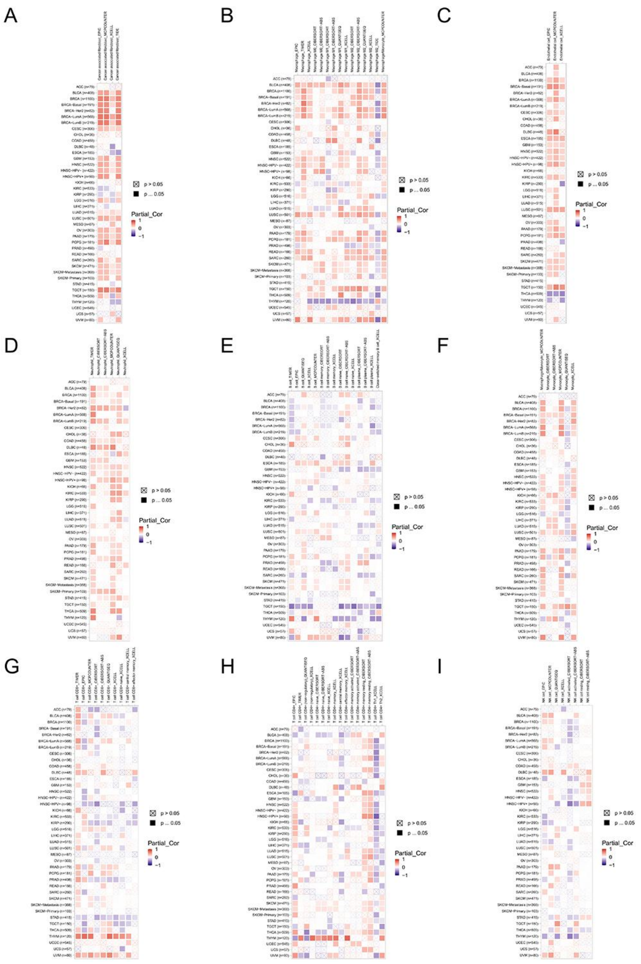

Figure S7. TIMER 2.0 database analysis of the correlation between DPP4 expression and immune cell (cancer-associated fibroblasts, macrophages, endothelial, neutrophils, B cells, monocyte, T cells CD8, T cells CD4, and NK cells) infiltration across pan-cancer types. (**A**) DPP4 expression was positively correlated with cancer-associated fibroblasts in BLCA, BRCA, CESC, GBM, HNSC, LGG, LUAD, LUSC, MESO, OV, PAAD, PCPG, SARC, SKCM, TGCT, THCA, and UVM. (**B**) DPP4 expression was positively correlated with macrophages in BLCA, BRCA, HNSC, LUSC, PAAD, PCPG, READ, SARC, SKCM, TGCT, and UVM, while it was negatively correlate in THYM. (**C**) DPP4 expression was positively correlated with endothelial cells in BRCA, CESC, DLBC, ESCA, GBM, HNSC, KIRC, LGG, LUSC, OV, PAAD, PCPG, SARC, SKCM, TGCT, while it was negatively correlated in THCA, THYM. (**D**) DPP4 expression was positively correlated with neutrophils in BLCA, BRCA, COAD, HNSC, KICH, KIRC, OV, PRAD, SKCM, and STAD. (**E**) DPP4 expression was negatively correlated with B cells in TGCT and THYM. (**F**) DPP4 expression was positively correlated with monocytes in BRCA, LUSC, PAAD, and TGCT. (**G**) DPP4 expression was positively correlated with T cells CD8 in THYM and UVM, while it was negatively correlated in KIRP and STAD. (**H**) DPP4 expression was positively correlated with T cells CD4 in THYM. (**I**) DPP4 expression was negatively correlated with NK cells in THYM.

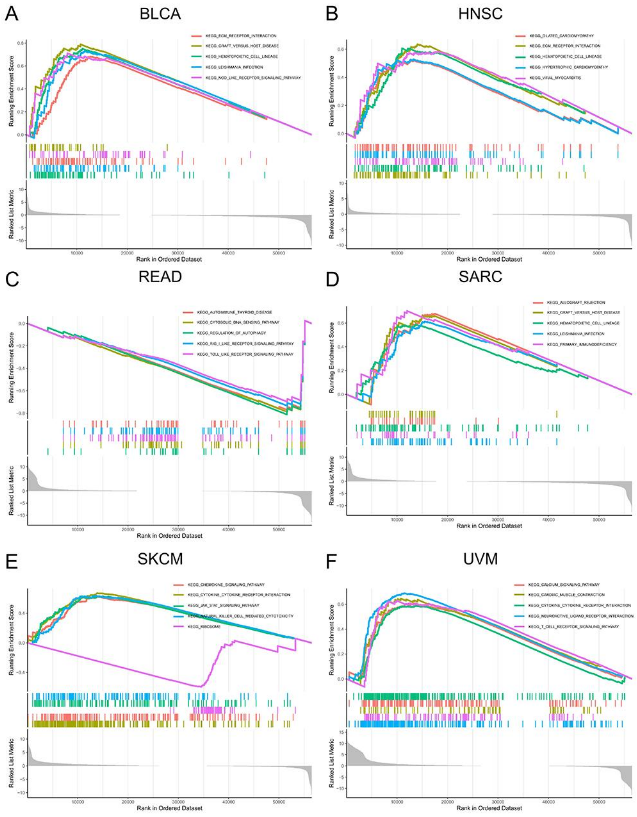

Figure S8. GSEA analysis of DPP4 in pan-cancer. (**A**) In BLCA, the top five KEGG up-regulated pathways were "ECM-receptor interaction", "graft-versus-host disease", "hematopoietic cell lineage", "leishmania infection", and "NOD-like receptor signaling pathway". (**B**) In HNSC, the top five KEGG up-regulated pathways were "dilated cardiomyopathy", "ECM-receptor interaction", "hematopoietic cell lineage", "hypertrophic cardiomyopathy", and "viral myocarditis". (**C**) In READ, the top five KEGG down-regulated pathways were "autoimmune thyroid disease", "cytosolic DNA-sensing pathway", "regulation of autophagy", "RIG-I-like receptor signaling pathway", and "Toll-like receptor signaling pathway". (**D**) In SARC, the top five KEGG up-regulated pathways were "allograft rejection", "graft-versus-host disease", "hematopoietic cell lineage", "leishmania infection", and "primary immunodeficiency". (**E**) In SKCM, the KEGG up-regulated pathways were "chemokine signaling pathway", "cytokine-cytokine receptor interaction", "JAK-STAT signaling pathway", and "natural killer cell mediated cytotoxicity", and the KEGG down-regulated pathway was "ribosome". (**F**) In UVM, the top five KEGG up-regulated pathways were "calcium signaling pathway", "cardiac muscle contraction", "cytokine-cytokine receptor interaction", "neuroactive ligand-receptor interaction", and "T cell receptor signaling pathway".

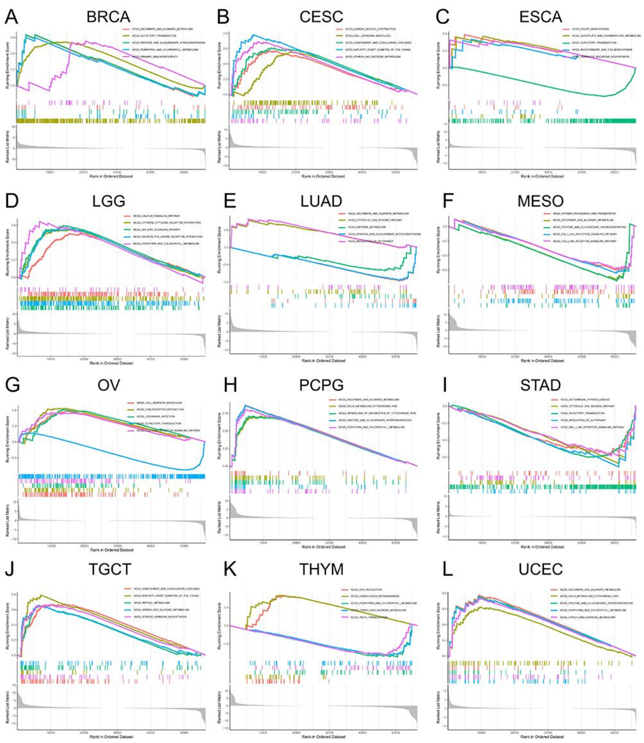

Figure S9. GSEA analysis of DPP4 in pan-cancer. (**A**) In BRCA, the top five KEGG up-regulated pathways were “ascorbate and aldarate metabolism”, “olfactory transduction”, “pentose and glucuronate interconversions”, “porphyrin and chlorophyll metabolism”, and “primary immunodeficiency”. (**B**) In CESC, the top five KEGG up-regulated pathways were “cardiac muscle contraction”, “cell adhesion molecules”, “complement and coagulation cascades”, “maturity onset diabetes of the young”, and “starch and sucrose metabolism”. (**C**) In ESCA, the KEGG up-regulated pathways were “folate biosynthesis”, “glyoxylate and dicarboxylate metabolism”, “pantothenate and CoA biosynthesis”, and “terpenoid backbone biosynthesis”, and the KEGG down-regulated pathway was “olfactory transduction”. (**D**) In LGG, the top five KEGG up-regulated pathways were “calcium signaling pathway”, “cytokine-cytokine receptor interaction”, “JAK-STAT signaling pathway”, “neuroactive ligand-receptor interaction”, and “porphyrin and chlorophyll metabolism”. (**E**) In LUAD, the KEGG up-regulated pathways were “regulation of autophagy” and “cytosolic DNA-sensing pathway”, and the KEGG down-regulated pathways were “ascorbate and aldarate metabolism”, “histidine metabolism”, and “pentose and glucuronate interconversions”. (**F**) In MESO, the top five KEGG down-regulated pathways were “antigen processing and presentation”, “ascorbate and aldarate metabolism”, “pentose and glucuronate interconversions”, “RIG-I-like receptor signaling pathway”, and “Toll-like receptor signaling pathway”. (**G**) In OV, the KEGG up-regulated pathways were “cell adhesion molecules”, “ECM-receptor interaction”, “leishmania infection”, and “Toll-like receptor signaling pathway”, and the KEGG down-regulated pathway was “olfactory transduction”. (**H**) In PCPG, the top five KEGG up-regulated pathways were “ascorbate and aldarate metabolism”, “drug metabolism cytochrome P450”, “metabolism of xenobiotics by cytochrome P450”, “pentose and glucuronate interconversions”, and “porphyrin and chlorophyll metabolism”. (**I**) In STAD, the top five KEGG down-regulated pathways were “autoimmune thyroid disease”, “cytosolic DNA-sensing pathway”, “olfactory transduction”, “regulation of autophagy”, and “RIG-I-like receptor signaling pathway”. (**J**) In TGCT, the top five KEGG up-regulated pathways were “complement and coagulation cascades”, “maturity onset diabetes of the young”, “retinol metabolism”, “starch and sucrose

metabolism”, and “steroid hormone biosynthesis”. (**K**) In THYM, the KEGG up-regulated pathways were “DNA replication” and “homologous recombination”, and the KEGG down-regulated pathways were “porphyrin and chlorophyll metabolism”, “starch and sucrose metabolism”, and “taste transduction”. (**L**) In UCEC, the top five KEGG up-regulated pathways were “ascorbate and aldarate metabolism”, “drug metabolism cytochrome P450”, “pentose and glucuronate interconversions”, “porphyrin and chlorophyll metabolism”, and “starch and sucrose metabolism”.

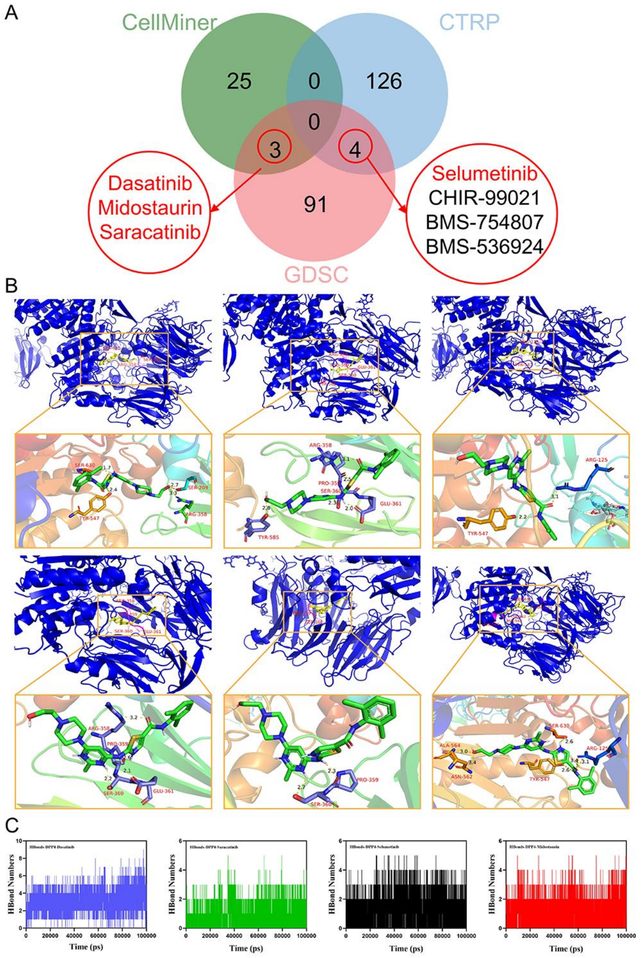

Figure S10. Molecular docking analysis. (**A**) Venn plot illustrating the intersection among results identified by Cellminer, CTRP, and GDSC. Dasatinib, Midostaurin, Saracatinib, and Selumetinib were selected for molecular docking for their lowest p-value in the correlation analysis. (**B**) Possible binding sites between DPP4 and dasatinib. **(C)** The number of hydrogen bonds between DPP4 and each small molecule. For the DPP4-Dasatinib complex, the bond count ranged from 0 to 9, with approximately 5 bonds present in most cases. For the DPP4-Saracatinib complex, the bond count ranged from 0 to 5, with approximately 3 bonds in most cases. For the DPP4-Selumetinib complex, the bond count ranged from 0 to 4, with approximately 2 bonds in most cases. For the DPP4-Midostaurin complex, the bond count ranged from 0 to 5, also with approximately 2 bonds present in most cases.

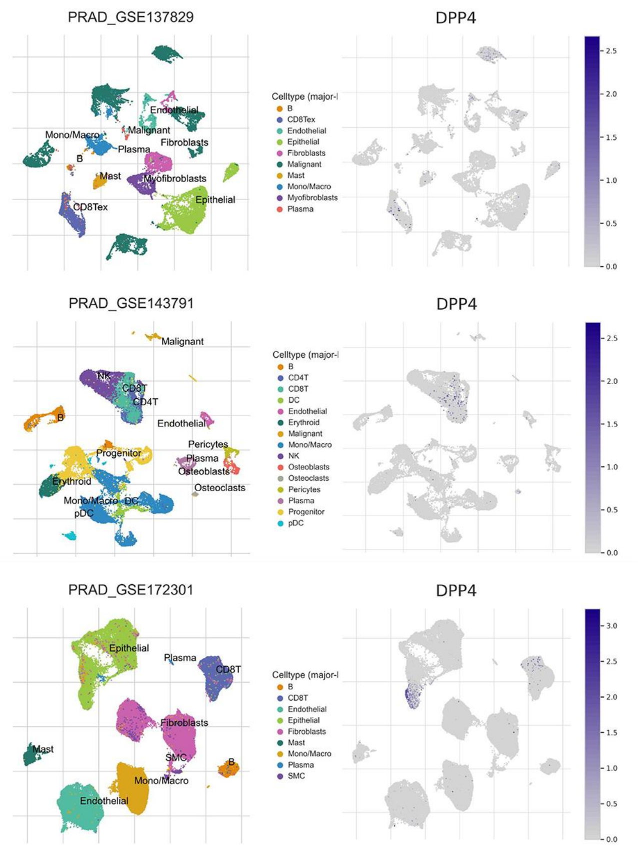

Figure S11. Single-cell sequencing analysis from TISCH in (**A**) GSE137829, (**B**) GSE143791, and (**C**) GSE172301. DPP4 was highly expressed in epithelial cells and CD8+ T cells.

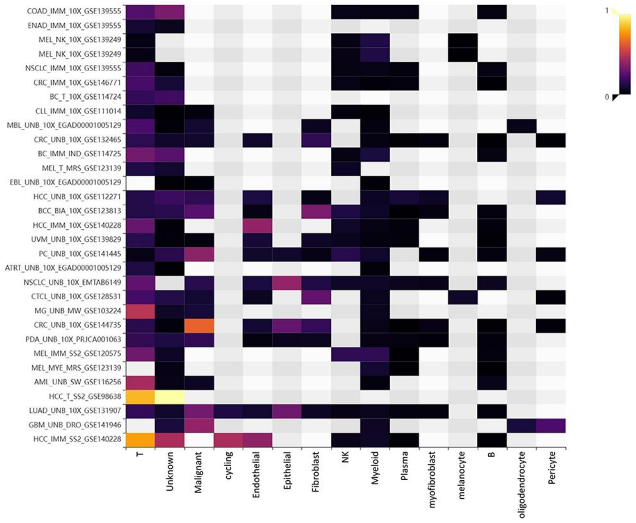

Figure S12. Single-cell sequencing analysis from IMMUcan database. IMMUcan SingleCell RNAseq Database showed that DPP4 expression was high in T cells in pan-cancer.

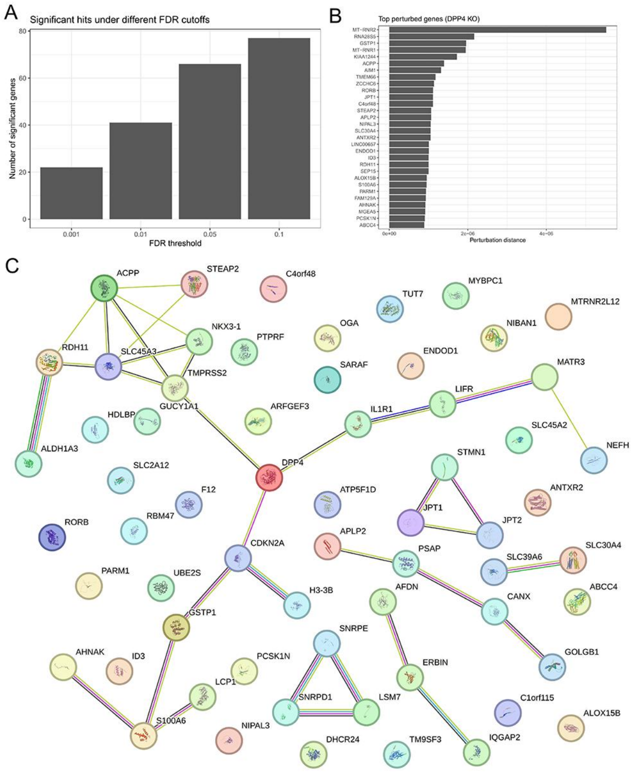

Figure S13. Transcriptomic perturbation landscape and protein-protein interaction network. (A) The number of significant genes under different FDR threshold in virtual KO. (B) Bar plot of top-ranked 30 genes (adjusted p < 0.05) affected by DPP4 knockout, based on perturbation distance. (**C**) PPI network of significantly perturbed genes following virtual KO of DPP4. *KO, Knockout; PPI, Protein-protein interaction.*

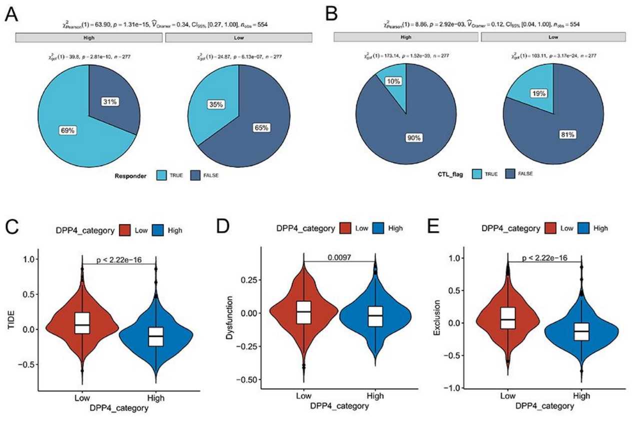

Figure S14. TIDE analysis of DPP4 in PRAD. Higher DPP4 expression was correlated with (**A**) higher response rate for immune checkpoint blockade therapy, (**B**) lower CTL infiltration, (**C**) lower TIDE score, (**D**) lower rate of tumor T cell dysfunction potential, and (**E**) lower rate of tumor T cell exclusion potential. *TIDE, Tumor Immune Dysfunction and Exclusion; CTL, Cytotoxic T Lymphocytes.*

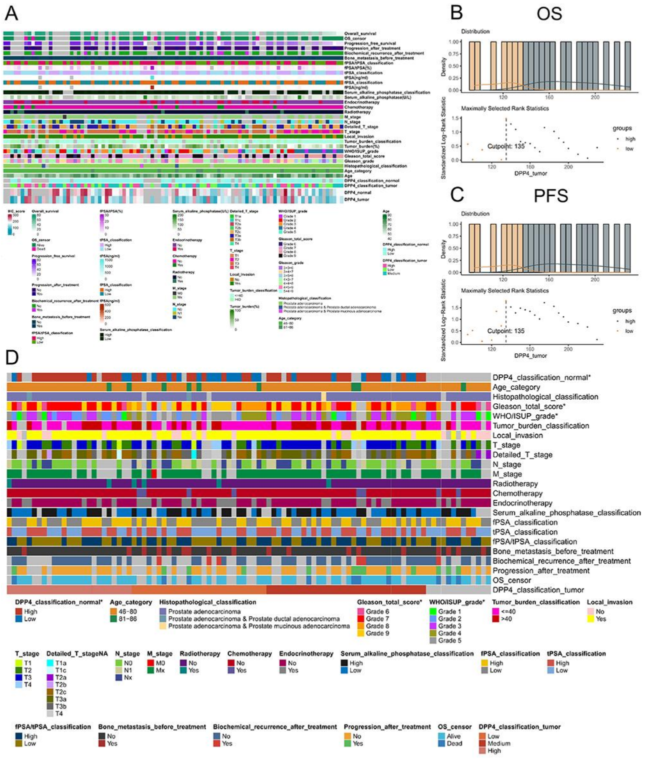

Figure S15. The cutoff point of IHC scores and the heatmap with Chi square analysis. (**A**) The heatmap illustrates the demographic and clinicopathological features of the cohort, comprising 97 prostate cancer patients. (**B**) For OS in prostate cancer, the cutoff point of DPP4 IHC scores was determined to be 135. (**C**) For PFS in prostate cancer, the cutoff point of DPP4 IHC scores was identified as 135. (**D**) The heatmap displaying the result of Chi square analysis. DPP4 expression was significantly correlated with Gleason total score ($p < 0.05$) and WHO/ISUP grade ($p < 0.05$). *IHC, Immunohistochemical; OS, Overall Survival; PFS, Progression-Free Survival; *$p < 0.05$, **$p < 0.01$, ***$p < 0.001$.*

Table S1 | The abbreviation of all 33 tumor types from TCGA.

Tumor	Abbreviation
ACC	Adrenocortical carcinoma
BLCA	Bladder Urothelial Carcinoma
BRCA	Breast invasive carcinoma
CESC	Cervical squamous cell carcinoma and endocervical adenocarcinoma
CHOL	Cholangiocarcinoma
COAD	Colon adenocarcinoma
DLBC	Lymphoid Neoplasm Diffuse Large B-cell Lymphoma
ESCA	Esophageal carcinoma
GBM	Glioblastoma multiforme
HNSC	Head and Neck squamous cell carcinoma
KICH	Kidney Chromophobe
KIRC	Kidney renal clear cell carcinoma
KIRP	Kidney renal papillary cell carcinoma

LAML	Acute Myeloid Leukemia
LGG	Brain Lower Grade Glioma
LIHC	Liver hepatocellular carcinoma
LUAD	Lung adenocarcinoma
LUSC	Lung squamous cell carcinoma
MESO	Mesothelioma
OV	Ovarian serous cystadenocarcinoma
PAAD	Pancreatic adenocarcinoma
PCPG	Pheochromocytoma and Paraganglioma
PRAD	Prostate adenocarcinoma
READ	Rectum adenocarcinoma
SARC	Sarcoma
SKCM	Skin Cutaneous Melanoma
STAD	Stomach adenocarcinoma
TGCT	Testicular Germ Cell Tumors
THCA	Thyroid carcinoma
THYM	Thymoma
UCEC	Uterine Corpus Endometrial Carcinoma
UCS	Uterine Carcinosarcoma
UVM	Uveal Melanoma

TCGA, The Cancer Genome Atlas.

Table S2 | The affinity, RMSD values and active site residues of each binding mode produced. RMSD was calculated compared to mode 1, which was the best predicted mode.

Ligand	Mode	Affinity (kcal/mol)	RMSD l.b. (Å)	RMSD u.b. (Å)	Active site residues
Dasatinib	1	-8.8	0.000	0.000	ARG-358, SER-209, SER-630
	2	-8.6	1.601	2.144	ARG-358, SER-209, SER-630, TYR-547
	3	-8.4	11.150	17.433	ARG-358, GLU-361, PRO-359, SER-360, TYR-585
	4	-8.2	3.509	11.639	ARG-125, TYR-547
	5	-8.2	11.385	17.475	ARG-358, GLU-361, PRO-359, SER-360
	6	-8.2	11.421	17.475	PRO-359, SER-360
	7	-8.2	3.676	11.512	ALA-564, ARG-125, ASN-562, TYR-547, SER-630
	8	-8.1	3.843	10.982	SER-630
Midostaurin	1	-10.2	0.000	0.000	GLN-553, SER-630, TYR-547, TYR-585
	2	-9.4	3.334	6.554	ARG-358, TYR-547

	3	-9.4	7.415	12.725	ARG-125, ASN-562, TYR-752
	4	-9.4	6.130	10.646	TYR-48
	5	-9.0	4.417	8.276	ARG-125
	6	-9.0	3.676	8.962	/
	7	-8.9	19.858	22.724	LYS-122, LYS-250
	8	-8.8	3.269	7.227	SER-209
Saracatinib	1	-9.2	0.000	0.000	ASN-562, GLY-741, LYS-554, TYR-547, TYR-752
	2	-9.2	2.383	8.939	HIS-740, LYS-554, SER-630, TYR-752
	3	-9.0	1.513	2.013	GLY-741, LYS-554, TYR-752
	4	-8.9	2.758	3.916	ASN-562, GLY-741, TYR-752
	5	-8.9	1.382	2.045	ASN-562, GLY-741, LYS-554, TYR-547, TYR-752
	6	-8.8	3.084	4.282	GLY-741, LYS-554, TYR-752
	7	-8.8	19.416	24.291	ASP-192, GLU-191, THR-129
	8	-8.7	3.730	4.679	ASN-562, TYR-752
Selumetinib	1	-7.9	0.000	0.000	ARG-125, SER-630, TYR-547, TYR-662
	2	-7.9	4.050	6.390	SER-630, TYR-547, TYR-666
	3	-7.6	6.982	9.799	LYS-554
	4	-7.5	9.351	12.071	ALA-564, TYR-48, TYR-752
	5	-7.5	3.412	5.926	SER-630, TYR-547, TYR-631, TYR-662
	6	-7.5	7.860	10.869	TYR-752, TRP-629
	7	-7.4	6.083	8.131	VAL-546, TRP-629, GLY-741
	8	-7.4	7.705	10.788	TYR-752

RMSD, Root Mean Square Deviation; l.b., lower bound; u.b., upper bound.
